# Genomic profiling of non-small cell lung cancer with the rare pulmonary lymphangitic carcinomatosis and clinical outcome of the exploratory anlotinib treatment

**DOI:** 10.3389/fonc.2022.992596

**Published:** 2022-10-17

**Authors:** Changqing Dong, Wanwan Cheng, Meiling Zhang, Si Li, Lele Zhao, Dongsheng Chen, Yong Qin, Mingzhe Xiao, Shencun Fang

**Affiliations:** ^1^ Department of Thoracic Surgery, Nanjing Chest hospital, The Affiliated Brain Hospital of Nanjing Medical University, Nanjing, China; ^2^ Department of Respiratory Medicine, Nanjing Chest hospital, The Affiliated Brain Hospital of Nanjing Medical University, Nanjing, China; ^3^ Department of Oncology, The First Affiliated Hospital of Nanjing Medical University, Nanjing, China; ^4^ Nanjing Simcere Medical Laboratory Science Co., Ltd, The State Key Laboratory of Translational Medicine and Innovative Drug Development, Jiangsu Simcere Diagnostics Co., Ltd, Nanjing, China

**Keywords:** pulmonary lymphangitic carcinomatosis (PLC), non-small cell lung cancer (NSCLC), genomic profiling, anlotinib, survival

## Abstract

**Background:**

To evaluate the potential treatment for patients with non-small cell lung cancer (NSCLC) and rare malignant pulmonary lymphangitis carcinomatosis (PLC), our study provided a genomic profile and clinical outcome of this group of patients.

**Methods:**

We retrospectively reviewed patients with NSCLC who developed PLC. The genomic alterations, tumor mutation burden (TMB), and microsatellite instability (MSI) based on DNA-based next-generation sequencing were reviewed and compared in a Chinese population with lung adenocarcinomas (Chinese-LUAD cohort). Clinical outcomes after exploratory anlotinib treatment and factors influencing survival are summarized.

**Results:**

A total of 564 patients with stage IV NSCLC were reviewed, and 39 patients with PLC were included. Genomic profiling of 17 adenocarcinoma patients with PLC (PLC-LUAD cohort) revealed *TP53*, *EGFR*, and *LRP1B* as the three most frequently altered genes. *EGFR* was less mutated in PLC-LUAD than Chinese-LUAD cohort of 778 patients (35.3% vs. 60.9%, *P = 0.043*). *BRIP1* was mutated more often in the PLC-LUAD cohort (11.8% vs. 1.8%, *P= 0.043*). Two patients presented with high tumor mutational burden (TMB-H, 10 mutations/MB). Combing alterations in the patient with squamous cell carcinoma, the most altered pathways of PLC included cell cycle/DNA damage, chromatin modification, the RTK/Ras/MAPK pathway and VEGF signaling changes. Fourteen of the participants received anlotinib treatment. The ORR and DCR were 57.1% and 92.9%, respectively. Patients achieved a median progression-free survival of 4.9 months and a median overall survival of 7 months. The adverse effects were manageable. In patients with adenocarcinoma, the mPFS (5.3 months vs. 2.6 months) and mOS (9.9 months vs. 4.5 months) were prolonged in patients receiving anlotinib treatment compared to those receiving other treatment strategies (*P < 0.05*).

**Conclusion:**

Patients with PLC in NSCLC demonstrated distinct genetic alterations. The results improve our understanding of the plausible genetic underpinnings of tumorigenesis in PLC and potential treatment strategies. Exploratory anlotinib treatment achieved considerable benefits and demonstrated manageable safety.

## Introduction

Pulmonary lymphangitic carcinomatosis (PLC) is caused by lymphatic spreading of metastatic cancer cells in the lungs. Among malignant tumors, PLC accounts for 6%–8% of intrathoracic metastases (1). Patients harboring this rare type of cancer often exhibit a poor prognosis due to delayed diagnosis, poor health conditions, and resistance to various therapies (2). Therefore, improving the understanding of PLC and exploring efficient treatments are urgent and important for these patients.

Lung cancer is the second most commonly reported primary tumor and is associated with the development of PLC (2). Recently, targeted therapy and immunotherapy have brought significant improvements in the survival rates of patients with NSCLC (3), but observations of the clinical and molecular characteristics of lung cancer with malignant PLC are still scarce. Genomic profiling of gene alterations could provide a comprehensive view of a patient’s targetable mutations. Simultaneously, alterations to the pathways involved in PLC might also indicate the mechanisms involved in the development of PLC and potential therapeutic strategies.

Researchers have reported few evaluations of the efficacy and safety of regimens for PLC due to its relatively low prevalence and dispersed locations of primary tumors. Isolated studies included the assessment of the remission of PLC and disease control with hormonal therapy (4), chemotherapy (5) (6), antiangiogenic drugs, including apatinib (7), regorafenib (8), bevacizumab (9), cetuximab (10), and gefitinib (11, 12), and immunotherapy (13). These highlighted potential treatment strategies for patients who develop PLC and studies with detailed descriptions are warranted to guide the management of patients. Anlotinib has recently been demonstrated to suppress lymphangiogenesis and lymphatic metastasis in mouse models of lung adenocarcinoma (14). A summary of its efficacy and safety in clinical practice would provide an important reference for patients considering anlotinib.

In this study, we retrospectively reviewed patients with NSCLC who developed PLC and illustrated the full picture of the patients’ clinical characteristics, genomic alterations, and clinical outcomes. The results of our study may provide a comprehensive understanding of NSCLC with PLC and offer potential treatment options for this group of patients. Our preliminary results were presented at the annual meeting of the American Society of Clinical Oncology in 2021 (15).

## Materials and methods

### Patients and data collection

**Figure 1:**
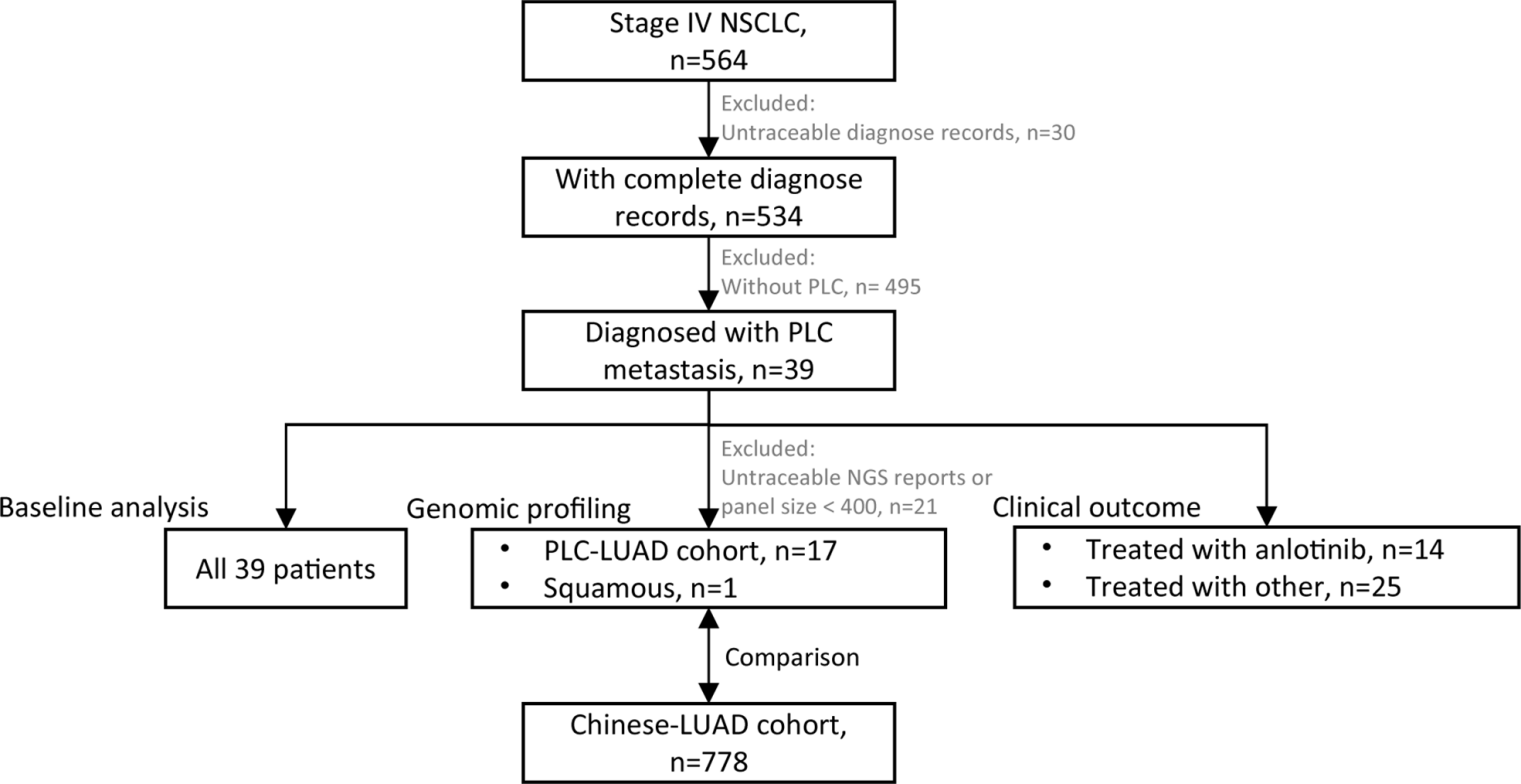
Flowchart of patient selection. 39 patients were enrolled for baseline analysis; 18 patients (17 of PLC-LUAD cohort, 1 squamous) with traceable DNA-based next generation sequencing (NGS, panel size > 400 genes) reports were enrolled for genomic profiling, and that of PLC-LUAD was compared to lung adenocarcinoma in Chinese population (Chinese-LUAD cohort); 14 patients treated with anlotinib and 25 with other treatment strategies were enrolled for clinical outcome evaluation.

We retrospectively reviewed patients who were treated at the Department of Respiratory Medicine of Nanjing Chest Hospital (The Affiliated Brain Hospital of Nanjing Medical University) from January 2018 to May 2021 (**Figure 1**). The inclusion criteria were metastatic or recurrent NSCLC (Stage IV) with a complete diagnosis report and confirmed metastasis of PLC.

The following information on the enrolled patients was collected for baseline analysis: age, sex, Karnofsky performance scale (KPS) score, smoking history, histological subtype, sites of metastatic disease, site of PLC, and mutation status of *EGFR*. The Research Ethics Committee of Nanjing Chest Hospital approved this study.

### Genomic profiling

Among all patients, 18 patients previously diagnosed with DNA-based next-generation sequencing (NGS) with a panel size of > 400 genes (see **Supplementary Table S1** for a list of the panels and genes) were enrolled for genomic analysis. The samples included 11 tumor tissues from biopsy specimens, seven plasma specimens, and one cerebrospinal fluid specimen, all of which were obtained before treatment. To illustrate the common genomic profile, 274 intersecting genes were identified (**Table S1**). Alteration types [single nucleotide variation (SNV)/insertion and deletion (InDel)/copy number variation (CNV)/intron/rearrangement] and the frequencies of the intersecting 274 genes, TMB (tumor mutation burden, nonsynonymous), and microsatellite instability (MSI) were reviewed.

Alterations found in 778 patients with lung adenocarcinoma in a Chinese population (Chinese-LUAD cohort) were extracted and compared to the alterations found in the PLC-LUAD cohort. From January 1, 2019, to December 31, 2020, tumor or plasma samples collected from the 778 patients with lung adenocarcinoma were analyzed with a panel-based NGS assay (539 genes) at the College of American Pathologists-certified laboratory in Jiangsu Simcere Diagnostics Co., Ltd. Genomic DNA (gDNA) of the sample tissues was extracted using a tissue sample DNA extraction kit (Kai Shuo). Genomic DNA from leucocytes and cell-free DNA (cfDNA) in plasma were extracted with a MagMAXTM DNA Multi-Sample Ultra kit (Thermo Fisher). Following the manufacturer’s instructions, extraction procedures were performed. The probe hybridization capture method was used for gene library construction. Commercial reagents and customized probes were used for library construction and hybridization capture. In brief, 15–200 ng of gDNA was sheared into 200–350 bp fragments with fragmentation enzymes. Base calls obtained with the Illumina NovaSeq6000 were converted to FASTQ files. The fastp tool (v.2.20.0) was used for adapter trimming and filtering low-quality bases (16). Duplicate reads obtained by PCR were excluded using Dedup with Error Correct. SNVs/InDels were called and annotated *via* VarDict (v.1.5.7) and InterVar (17, 18), and then, the variants were filtered against common SNPs in public databases, including the 1,000 Genome Project (Aug 2015) and Exome Aggregation Consortium Browser28 (v.0.3). Next, CNVs and fusions were analyzed with a CNV kit (dx1.1) and factera (v1.4.4) (19) (20), respectively. Alterations in SNVs, InDels, CNVs, introns, and rearrangements were extracted from the Chinese-LUAD cohort and compared with the LUAD-PLC cohort.

### Treatment strategy and follow-ups

Fourteen patients received anlotinib-based treatment (the Anlotinib group), and the other 25 patients received other stratifications based on clinical practice (the Other group) (baseline comparison in **Table S2**). The response, survival, and safety data for patients treated with anlotinib were assessed. Anlotinib was administered orally at a dose of 12 mg daily. Responses to anlotinib were evaluated after 3 to 4 weeks of initial administration and during the regular follow-up visits (every 1–2 months), according to the Response Evaluation Criteria in Solid Tumors (RECIST, v1.1). Next, the progression-free survival (PFS) rate, overall survival (OS) rate, and objective response rate (ORR) were collected. The toxicities were assessed according to the Common Terminology Criteria for Adverse Events. PFS and OS were collected for the Other group.

### Statistical analysis

Fisher’s Exact Test and Wilcoxon rank sum test were used for comparisons of categorical and continuous variables, respectively. Survival data were presented in Kaplan-Meier curves and compared using the log-rank test. The impact of factors on PFS and OS rates were evaluated using univariable COX regression analysis. All the statistical analyses were conducted with R version 4.0.3 (http://www.r-project.org). A two-sided *P < 0.05* was considered statistically significant.

## Results

### Demographics of the enrolled patients

A total of 564 patients with stage IV NSCLC were reviewed, and 39 patients were eventually enrolled with confirmed metastasis of PLC (**Figure 1**). The patients’ baseline characteristics are summarized in **Table 1**. The median age was 63 years, with a range of 30–80 years. Twenty-six (66.7%) patients were male. The median (range) KPS was 70 (range 40–90). Twenty-one (53.8%) patients presented with a history of smoking, and 35 (89.7%) were diagnosed with adenocarcinoma. The most frequent metastatic sites were the bones (25; 64.1%) and lungs (18; 46.2%). PLC lesions were predominantly observed in the right lung (34; 87.3%).

**Table 1:** Baseline characteristics.

Variables	All patients	Genomic profling	Treated with anlotinb
	n (%)/median (range)	n (%)/median (range)	n (%)/median (range)
**No. of Patient**	39 (100%)	18 (100%)	14 (100%)
**Age (years)**	63 (30-80)	63 (30-80)	63 (34-79)
**Sex (male)**	26 (66.7%)	10 (55.6%)	9 (64.3%)
**Baseline KPS**	70 (40-90)	65 (50-90)	65 (40-90)
**Smoking history (former)**	21 (53.8%)	8 (44.4%)	5 (35.7%)
**Histological subtype**			
**Adenocarcinoma**	35 (89.7%)	17 (94.4%)	12 (85.7%)
**Squamous**	4 (10.3%)	1 (5.6%)	2 (14.3%)
**EGFR (mutant)**	14 (35.9%)	6 (33.3%)	4 (28.6%)
**Sites of other metastasis**			
**Bone**	25 (64.1%)	12 (66.7%)	5 (35.7%)
**Lung**	18 (46.2%)	8 (44.4%)	8 (57.1%)
**Pleura**	15 (38.5%)	5 (27.8%)	10 (71.4%)
**Brain**	14 (35.9%)	6 (33.3%)	4 (28.6%)
**Others**	9 (23.1%)	2 (11.1%)	2 (14.3%)
**Side of PLC**			
**Right**	34 (87.2%)	15 (83.3%0	11 (78.6%)
**Bilateral**	4 (10.3%)	3 (16.7%)	2 (14.3%)
**Left**	1 (2.6%)	0	1 (7.1%)

### Genomic profile analysis

**Figure 2:**
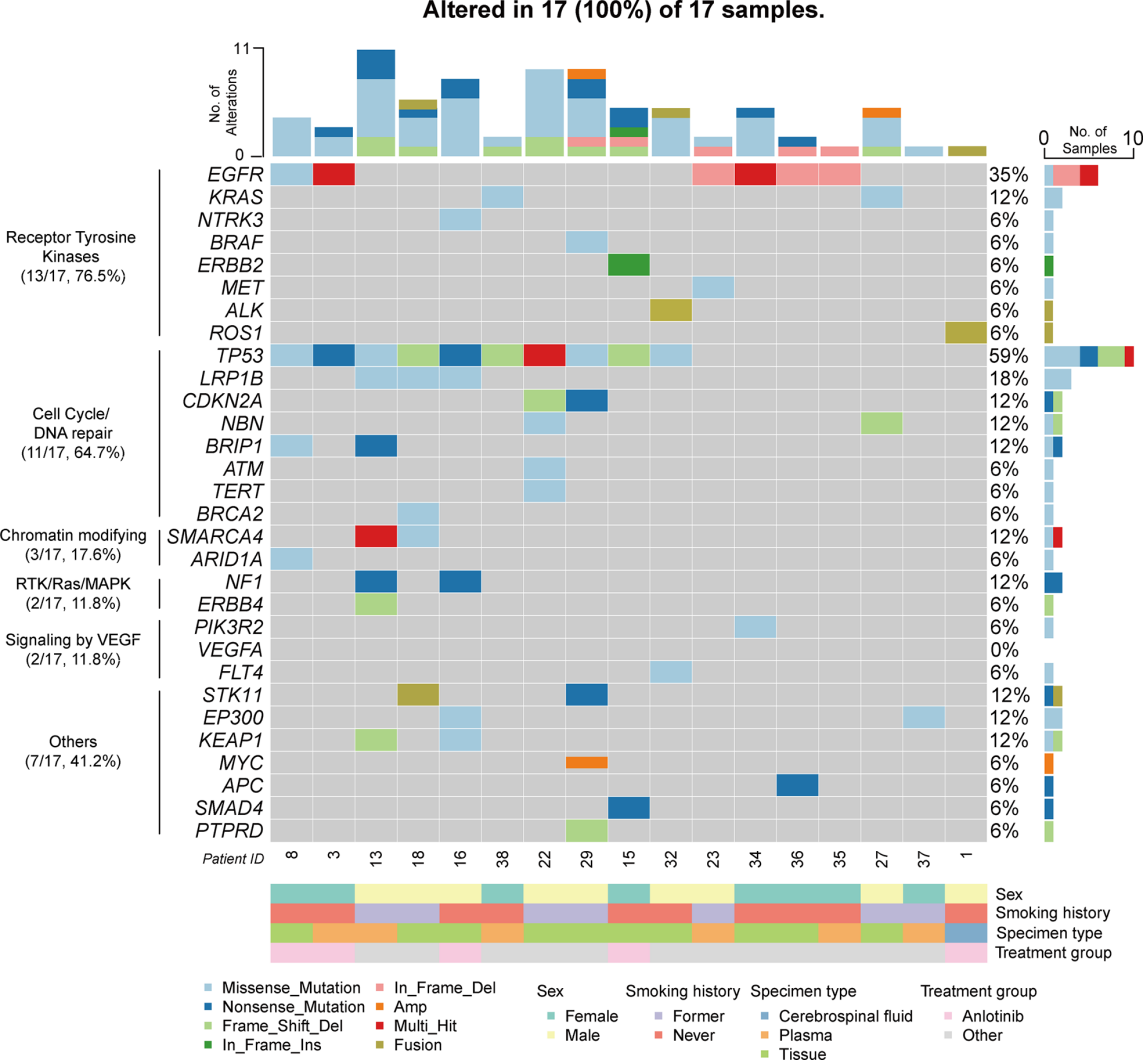
Genomic profiling of PLC-LUAD cohort (17 patients). Alterations presented here included single nucleotide variation/insertion and deletion/copy number variation/fusion. Top 30 altered genes were illustrated.

Eighteen patients were enrolled for genomic profiling (**Table 1**), and the landscape of 17 patients in PLC-LUAD is given in **Figure 2**. Overall, 80 alterations were discovered in PLC-LUAD cohort. *TP53* (10/17; 58.8%), *EGFR* (6/17; 35.3%), and *LRP1B* (3/17; 17.6%) were the most commonly altered genes. A total of 76.5% of patients harbored driver gene mutations, including those in *EGFR* (35.3%), *KRAS* (11.8%), *NTRK3* (5.9%), *BRAF* (5.9%), *ERBB2* (5.9%), and *MET* (5.9%), as well as fusions in *ALK* (5.9%) and *ROS1* (5.9%). Compared to the alterations in the Chinese-LUAD cohort (**Figure S1**), mutations in *EGFR* were less often observed in patients with PLC (35.3% vs. 60.9%; *P = 0.043*). Mutations of the *BRIP1* gene were more commonly observed in PLC (11.8% vs. 1.8%, *P = 0.043*). The remaining most frequently altered genes in the PLC-LUAD cohort were involved in the cell cycle/DNA damage pathway (64.7%), chromatin modifying pathway (17.6%), RTK/Ras/MAPK pathway (11.8%), and VEGF signaling pathway (11.8%). One patient with squamous cell carcinoma (Patient 33) harbored alterations in *TP53*, *LRP1B*, *ATM* and *VEGFA*, with a TMB of 2.87 mutations/MB and MSI-S.

The mean and median TMB of the PLC-LUAD cohort were 5.2 mutations/MB and 3.8 mutations/MB, respectively. Two patients (patients 13 and 16; 11.8%) demonstrated a TMB ≥ 10 mutations/MB (which was TMB-H). MSI was available in 15 patients, and none of these were considered microsatellite instability-high (MSI-H). The frequencies of TMB-H and MSI-H were comparable to those of the Chinese-LUAD cohort (TMB-H, 10%; MSI-H, 0.3%; *P > 0.5*).

### Clinical outcomes

**Figure 3:**
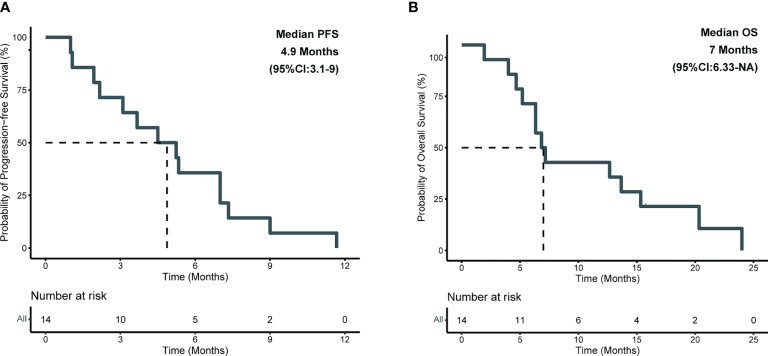
Kaplan-Meier survival analysis in anlotinib group. **(A)** Plots of progression-free survival (PFS); **(B)** Plots of overall survival (OS).

**Figure 4:**
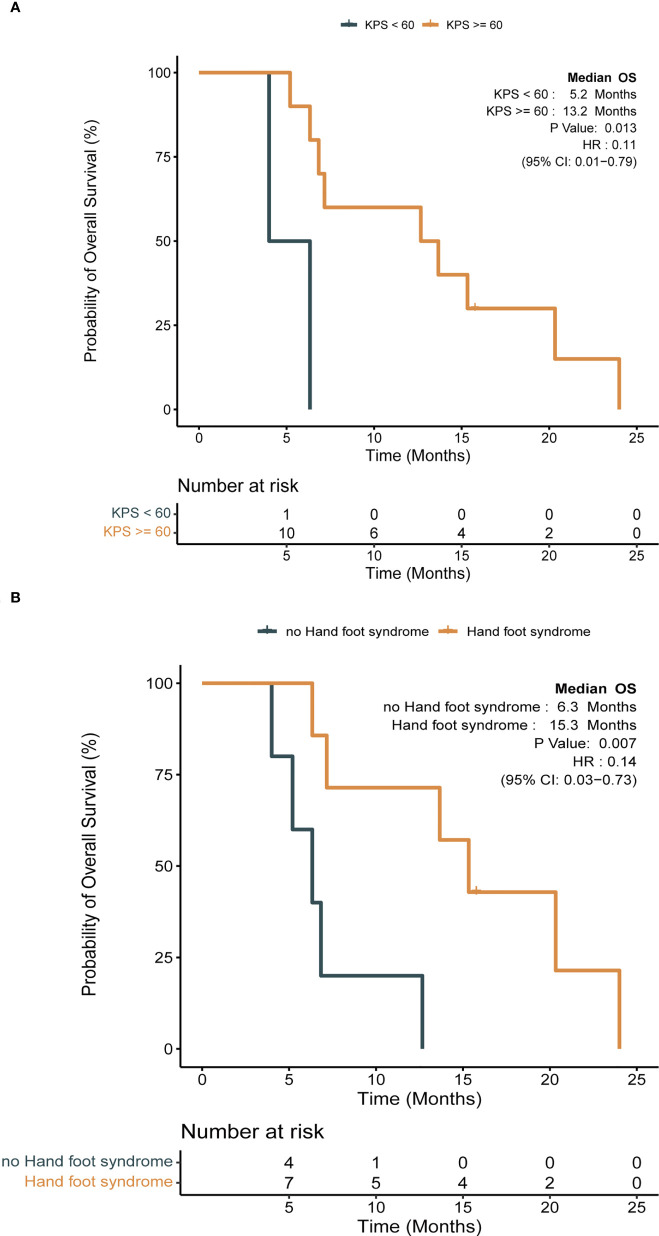
Impact of clinical factors on survival with Kaplan-Meier survival analysis in anlotinib group (log-rank test, *P < 0.05*). **(A)** Impact of Karnofsky performance scale (KPS) on OS; **(B)** impact of hand and foot syndrome (HFS) on OS.

Detailed baseline and clinical outcomes in the Anlotinib group are shown in **Table 2**. In all patients, the objective response rate (ORR) was 57.1% (8/14), and the disease control rate was 92.9% (13/14). The median PFS (mPFS) was 4.9 months, and the median OS (mOS) was 7 months (**Figures 3 A, B**). More specifically, patients with adenocarcinoma and squamous cell carcinoma had a mPFS of 5.3 months and 1.5 months, respectively (**Figure S2A**). The mOS of these patients were 9.9 months and 3.3 months, respectively (**Figure S2B**). The results of a univariable Cox regression analysis in 12 patients with adenocarcinoma (**Table 3**) revealed that patients with a favorable KPS (≥ 60) demonstrated longer mOS [13.2 months vs. 5.2 months, *P = 0.013*; (**Table 3**; **Figure 4A**)].

**Table 2:** Clinical characteristics and outcome of 14 patients treated with anlotinib.

Patient ID	Age (yrs.)	Sex	Smoking history	KPS	Histological type	EGFR (Mutant/Wildtype)	Enrolled in genomic profiling	Line	Response	PFS(months)	OS(months)
**1**	34	M	Never	80	Aden.	Wildtype	Yes	≥ 3	PR	11.7	12.7
**2**	72	F	Never	50	Aden.	Wildtype	No	≥ 3	PR	3.7	6.3
**3**	69	F	Never	70	Aden.	Mutant	Yes	≥ 3	PR	5.3	6.8
**4**	66	M	Never	50	Aden.	Wildtype	No	≥ 3	SD	2.2	4.0
**6**	65	M	Former	70	Aden.	Wildtype	No	2	PD	1.1	7.2
**8**	62	F	Never	90	Aden.	Mutant	Yes	≥ 3	PR	9.0	24.0
**9**	55	M	Former	50	Squa.	Wildtype	No	≥ 3	SD	1.5	4.7
**10**	63	M	Never	60	Aden.	Mutant	No	≥ 3	PR	3.1	5.2
**11**	42	M	Former	40	Squa.	Wildtype	No	≥ 3	SD	1.9	1.9
**12**	79	F	Never	70	Aden.	Wildtype	No	2	PR	7.3	20.3
**14**	63	M	Former	80	Aden.	Wildtype	No	≥ 3	SD	7.0	13.7
**15**	46	F	Never	60	Aden.	Wildtype	Yes	≥ 3	SD	4.5	6.3
**16**	66	M	Never	80	Aden.	Wildtype	Yes	≥ 3	PR	5.2	15.3
**19**	57	M	Former	60	Aden.	Mutant	No	2	PR	7.0	Alive (> 15)

M, male; F, female; Aden., adenocarcinoma; Squa., squamous; SD, stable disease; PR, partial response; PD, progressive disease.

**Table 3:** Results of univariable COX regression analysis of risk factors for PFS and OS in 12 patients with adenocarcinoma treated with anlotinib.

Variables	PFS		OS	
	HR [95% CI]	*P*	HR [95% CI]	*P*
Age (**≥** 64 years)	2.63 [0.72, 9.56]	0.142	1.76 [0.49, 6.29]	0.383
Sex (male)	1.17 [0.35, 3.88]	0.797	1.41 [0.35, 5.71]	0.629
KPS (**≥** 60)	0.16 [0.02, 1.14]	0.068	**0.11 [0.01, 0.79]**	**0.029**
*EGFR* (mutant)	0.87 [0.25, 3.01]	0.832	0.41 [0.09, 1.98]	0.269
*TP53* (mutant)	1.65 [0.39, 7.04]	0.454	0.45 [0.06, 3.53]	0.290
Smoking (former)	1.41 [0.29, 5.42]	0.710	0.58 [0.09, 2.44]	0.508
Strategy (combined)	3.48 [0.86, 14.14]	0.081	2.44 [0.62, 9.59]	0.202
Hand and foot syndrome	0.95 [0.27, 3.32]	0.940	**0.14 [0.03, 0.73]**	**0.020**
Diarrhea	0.63 [0.16, 2.42]	0.502	0.50 [0.10, 2.39]	0.385
Fatigue	2.50 [0.63, 9.85]	0.191	2.05 [0.52, 7.99]	0.303

HR and P values in bold indicated results of significant impact on OS (P<0.05).

Among 14 patients, the most frequently reported AEs were hypertension (10; 71.4%), hand foot syndrome (7; 50.0%) (HFS), fatigue (6; 42.9%), diarrhea (5; 35.7%), and hoarseness (3; 21.4%). An event of grade 3 was reported in 1 patient (7.1%) who presented with hypertension, and he continued treatment by reducing the dosage to 10 mg/day. Patients who presented with HFS during anlotinib treatment tended to demonstrate superior mOS [15.3 months vs. 6.3 months, *P = 0.007*; (**Table 3**; **Figure 4B**)].

Compared to the Other group of patients with adenocarcinoma, those in the Anlotinib group showed prolonged mPFS and mOS (mPFS, 5.3 months vs. 2.6 months, *P = 0.032*; mOS, 9.9 months vs. 4.5 months, *P = 0.005*; **Figure S3**). There were four patients with squamous cell carcinoma in total (Anlotinib group, n = 2; Other group, n = 2). The PFS was 1 and 1.93 months in the Anlotinib group and 1.4 and 3.3 months in the Other group; the OS was 1.93 and 4.67 months (Anlotinib group) and 5.5 and 6 months (Other group), respectively (**Table 2**; **Supplementary Data of Clinical and Treatment**). Comparisons in patients with squamous cell carcinoma were not performed due to limited sample size.

### Salvage treatment with anlotinib in a patient with PLC

**Figure 5:**
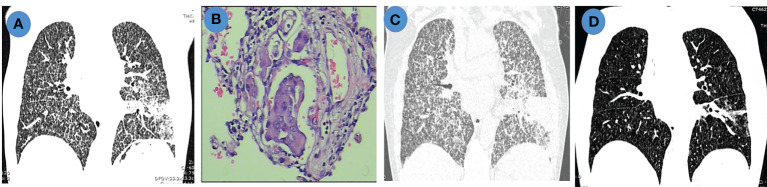
Chest imaging and pathological features in a patient with PLC. **(A)**. Chest CT scan shows pulmonary mass with diffuse PLC before chemotherapy; **(B)**. Hematoxylin and eosin (H&E) staining (200×) of lymphatic tumor embolus; **(C)**. Chest CT scan shows progressive disease (PD) after chemotherapy; **(D)**. Chest CT scan shows partial remission (PR) after anlotinib treatment.

A 79-year-old female nonsmoker (patient 12) was diagnosed with lung adenocarcinoma, and chest computed tomography (CT) revealed a mass in the left inferior lobe with diffuse carcinomatous lymphangitis in both lungs (**Figure 5A**). A microscopic image that could be used for making a pathological diagnosis of PLC is presented **Figure 5B**. DNA sequencing of primary tumor specimens revealed a *TP53* mutation. Then, the patient was treated with pemetrexed plus carboplatin chemotherapy as the first-line treatment. One week after chemotherapy, the patient developed refractory respiratory failure, accompanied by rapid progression of PLC in both lungs (**Figure 5C**). Then, anlotinib was initiated as salvage treatment. Dyspnea was significantly relieved after 3 days of treatment. The PLC in both lungs virtually disappeared after 1 month of treatment with anlotinib (**Figure 5D**), and the efficacy was evaluated as a partial response (PR). Furthermore, a good response in PLC was maintained for more than 7 months.

## Discussion

PLC is a rare type of intrathoracic metastasis of malignant tumors that leads to a poor prognosis. Our study aimed to provide full clinical and genomic profiles for patients with NSCLC and PLC, and we evaluated the outcomes of anlotinib treatment in clinical practice.

In our study, 7.3% (39/534) of patients with metastatic NSCLC were diagnosed with PLC. This confirms its low incidence, as reported previously (2). The molecular features of these patients have not been reviewed previously. Our genomic profiling revealed that most patients harbored driver gene alterations (76.5%; **Figure 2**), and 11.8% of the patients presented with TMB-H. These patients might benefit from targeted therapy and immunotherapy. Among the mutations in the driver genes, *EGFR* mutations were observed less often in the PLC-LUAD cohort, leaving fewer chances to benefit from *EGFR* TKIs in these patients. *BRCA1 interacting protein C-terminal helicase 1 (BRIP1)* mutations occurred more frequently in the cohort in our study compared with the Chinese-LUAD cohort. *BRIP1* is a homologous recombination-related gene, and previous reports included *BRIP1* as a risk gene for ovarian cancer (21). Additionally, *BRIP1* promotes cell proliferation and invasion in breast cancer (22). Next, in small-cell lung cancer, a patient harboring a germline mutation of *BRIP1* achieved a notable disease response to DNA repair-targeted therapies (23). These may present an opportunity for therapeutic interventions, similar to other HR defects for patients with *BRIP1* mutations. Additionally, *BRIP1* tends to be coexpressed with PD-L1 in lung adenocarcinoma (24). It mainly participates in the immune response and cell activation. Additionally, this might provide complementary biomarkers for predicting responses to PD-1/PD-L1 inhibitors.

Of note, VEGF signaling (through the *VEGFA*, *FLT4*, and *PIK3R2* genes) was one of the most frequently altered pathways in the PLC-LUAD cohort (11.8%, 5% in the Chinese-LUAD cohort, *P = 0.22*), and the patients with squamous cell carcinoma also harbored mutations in *VEGFA*. VEGF signaling is best known for its involvement in the development and maintenance of the blood and lymphatic vascular systems (25). Specifically, growth factor-C (VEGFC), whose receptor is encoded by *FLT4*, promotes the intralymphatic spread of metastases in the lung, thus inducing lymphangitic carcinomatosis (25–27). These results could provide important explanations for the development of PLC in our patients and, more importantly, suggest potential therapeutic approaches. Similarly, in a study of lung adenocarcinoma mouse models (14), anlotinib inhibited the growth and migration of human lymphatic endothelial cells and lymphangiogenesis *in vitro* and *in vivo*, probably through the inactivation of VEGFR-3 phosphorylation (28). This study indicated that anlotinib may be a promising treatment for PLC; however, its clinical outcomes still require systematic evaluation.

In this study, anlotinib had a mPFS of 4.9 months and a mOS of 7 months (**Figure 3**). More specifically, patients with adenocarcinoma achieved a mPFS of 5.3 months and a mOS of 9.9 months (**Figure S2**). Compared to the median survival of 2 months between symptom identification and death in the review by Klimek et al. (2), anlotinib showed preliminary improvements in survival benefits. This was also indicated by the improvements in the mPFS and mOS in the Anlotinib group compared to those in the Other group (**Figure S3**). Moreover, after reviewing previously reported cases (11–13, 29, 30), we calculated an ORR of 50% in gefitinib-treated patients with *EGFR* mutations (12), which was 100% in our study (**Table 2**). Apatinib-based treatment was reported to demonstrate a DCR of 69.2%, mPFS of 5.2 months, and mOS of 10.2 months (7) when not distinguishing patients’ baseline characteristics, treatment lines and strategies. A more detailed description might be important when evaluating the efficacy and treatment adoption for patients. In our results, a superior survival benefit was observed in patients with favorable KPS (mOS, 13.2 months), which was not inferior to patients treated with anlotinib with third-line treatment in the ALTER0303 study (PS, 0-1; mPFS, 5.4 months; mOS, 9.6 months) (31). This highlighted the benefit of anlotinib. Furthermore, we observed significantly longer OS (15.3 vs. 6.3 months, *P = 0.007*) in patients with HFS, which was in line with the ALTER0303 study (32). Inhibition of the VEGF pathway is widely discussed as the cause of HFS (33, 34), but the pathophysiology needs to be clarified. In summary, anlotinib might a potential regimen for treating PLC, especially in those patients with favorable KPS. The occurrence of HFS during treatment might predict superior efficacy.

Certainly, this study has intrinsic limitations. In this retrospective analysis, the samples collected and sequencing platforms were heterogeneous, which might introduce information bias on the results of genomic alterations. Furthermore, due to the lack of sequencing results in patients without PLC, we compared the profile of the PLC cohort with the whole population (LUAD cohort). This might have merged the difference between patients with and without PLC. Additionally, a larger sample size study might illustrate the alteration landscape more accurately. The limited number of evaluated patients and missing date due to the retrospective design also made it challenging to provide a comprehensive assessment of the clinical outcome of anlotinib and other strategies. This needs further verification.

## Conclusions

In conclusion, our study provided preliminary clinical and genomic profiles for NSCLC with PLC. The landscape of common driver genes revealed that most patients were candidates for targeted therapy. Analysis of the TMB and distinct mutations shed light on potential immunotherapy approaches and might simultaneously enhance the understanding of the tumorigenesis of PLC. Exploratory anlotinib treatment achieved considerable benefits and manageable safety. Further studies are warranted to validate these findings.

## Data availability statement

The datasets presented in this study can be found in online repositories. The names of the repository/repositories and accession number(s) can be found in the article/**Supplementary Material**.

## Ethics statement

The studies involving human participants were reviewed and approved by The Research Ethics Committee of Nanjing Chest Hospital. The patients/participants provided their written informed consent to participate in this study. Written informed consent was obtained from the individual(s) for the publication of any potentially identifiable images or data included in this article.

## Author contributions

Conception and design: SF. Administrative support: MX, YQ. Provision of study materials or patients: CD, WC, MZ. Data analysis and interpretation: SL, LZ. All authors contributed to the article and approved the submitted version.

## Funding

This study was supported by the “Six one projects” in Jiangsu Province (LGY2019006).

## Acknowledgments

We thank Qin Zhang, Yaqing Wu, Yangyang Yu, and Chuang Qi from Jiangsu Simcere Diagnostics for their kindly assistance.

## Conflict of interest

Authors SL, LZ, DC, YQ and MX were employed by the company Jiangsu Simcere Diagnostics Co., Ltd.

The remaining authors declare that the research was conducted in the absence of any commercial or financial relationships that could be construed as a potential conflict of interest.

## Publisher’s note

All claims expressed in this article are solely those of the authors and do not necessarily represent those of their affiliated organizations, or those of the publisher, the editors and the reviewers. Any product that may be evaluated in this article, or claim that may be made by its manufacturer, is not guaranteed or endorsed by the publisher.

## Supplementary material

Supplementary Figure 1 Genomic profiling of Chinese-LUAD cohort (778 patients). Alterations presented here included single nucleotide variation/insertion and deletion/copy number variation, etc.. Genes were listed according to the 30 top altered genes in PLC-LUAD cohort.

Supplementary Figure 2 Kaplan-Meier survival analysis in patients with adenocarcinoma and squamous treated by anlotinib. **(A)** Plots of progression-free survival (PFS); **(B)** Plots of overall survival (OS).

Supplementary Figure 3 Kaplan-Meier survival analysis and comparison of survival between group of anlotinib and other in patients with adenocarcinoma (log-rank test, *P < 0.05*). **(A)** Plots of progression-free survival (PFS); **(B)** Plots of overall survival (OS).
