# Supplementary figures and images for: Genomic profiling of non-small cell lung cancer with the rare pulmonary lymphangitic carcinomatosis and clinical outcome of the exploratory anlotinib treatment
Source: Front Oncol. 2022 Oct 17;12:992596. doi: 10.3389/fonc.2022.992596 (PMC9620420; doi:10.3389/fonc.2022.992596)

Altered in 744 (95.63%) of 778 samples.

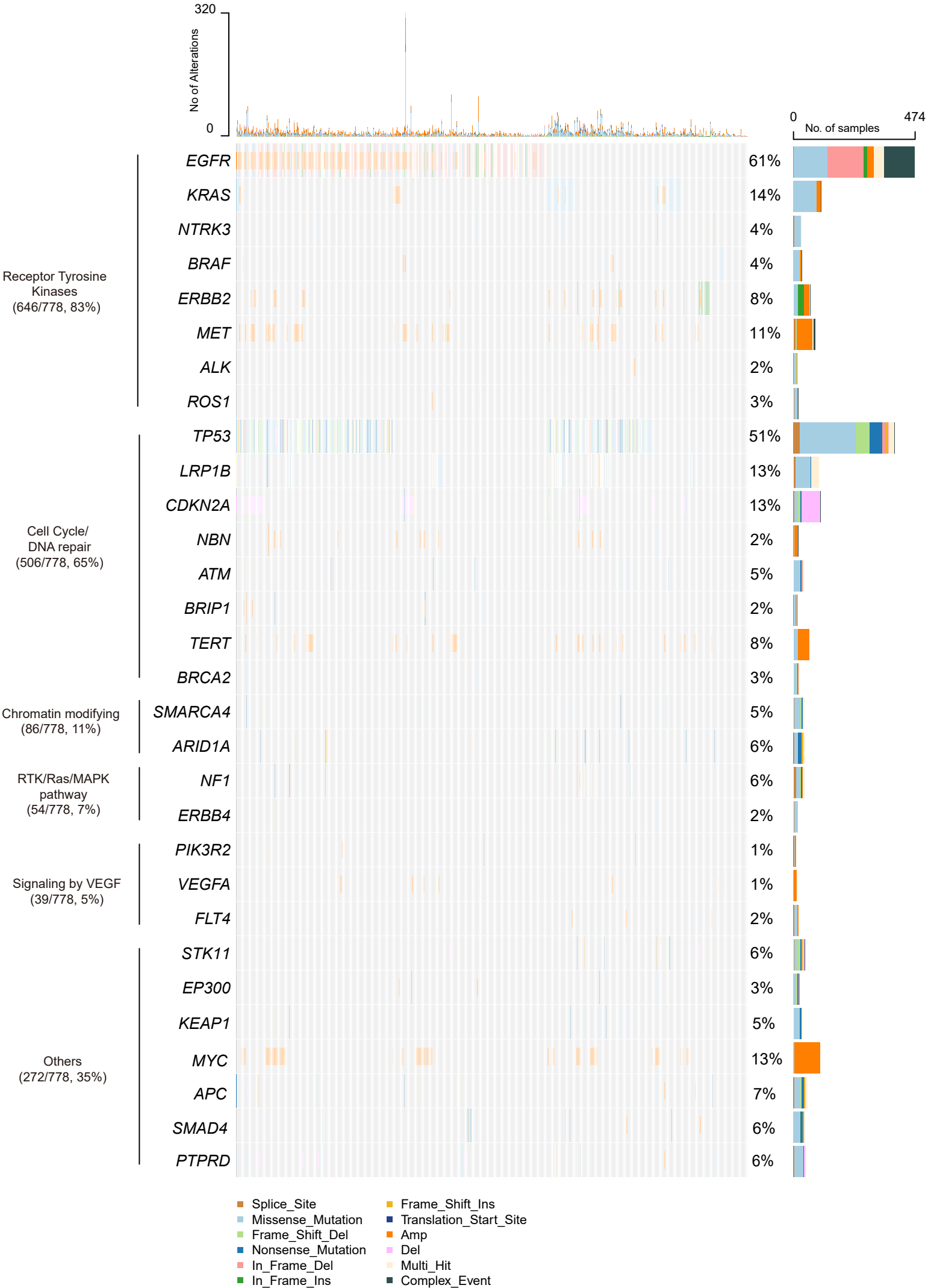

Supplement: Supplementary Figure 1 — Genomic profiling of Chinese-LUAD cohort (778 patients). Alterations presented here included single nucleotide variation/insertion and deletion/copy number variation, etc.. Genes were listed according to the 30 top altered genes in PLC-LUAD cohort. [file DataSheet_1.pdf]

(A)

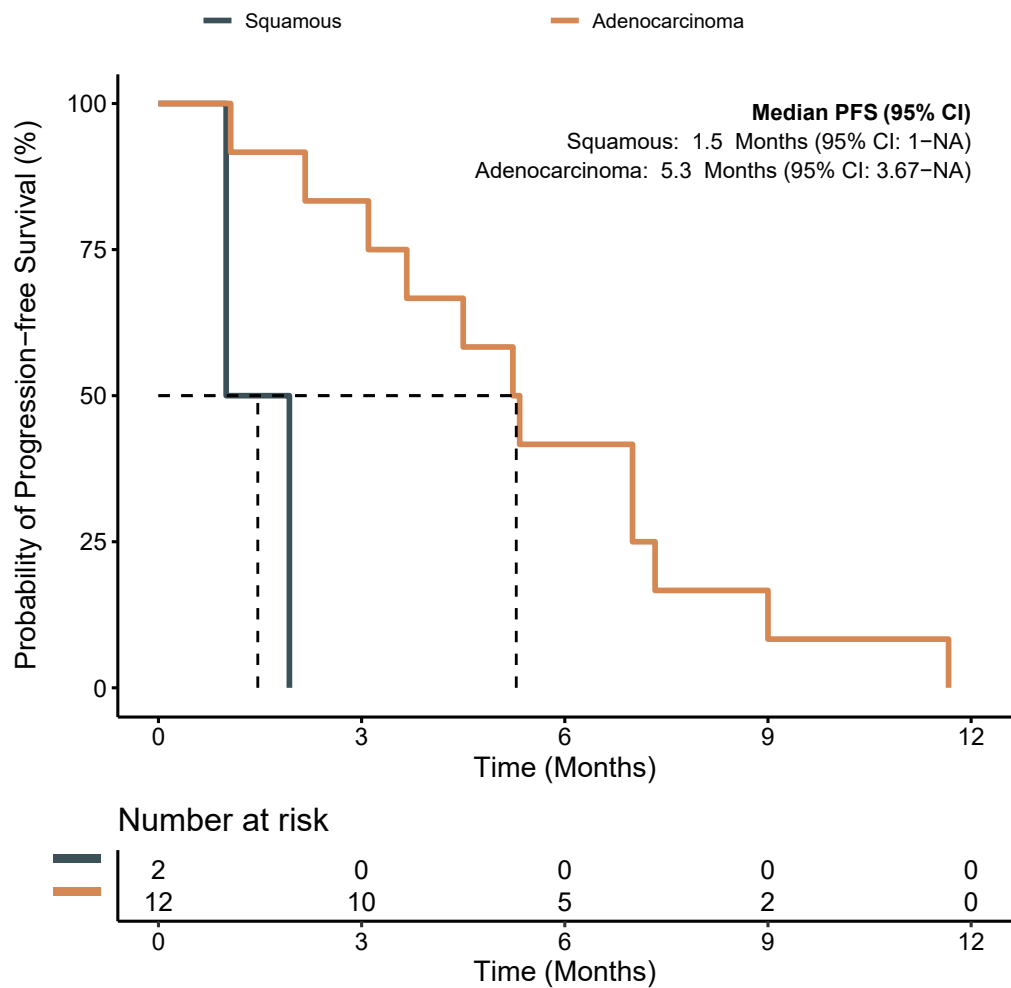

(B)

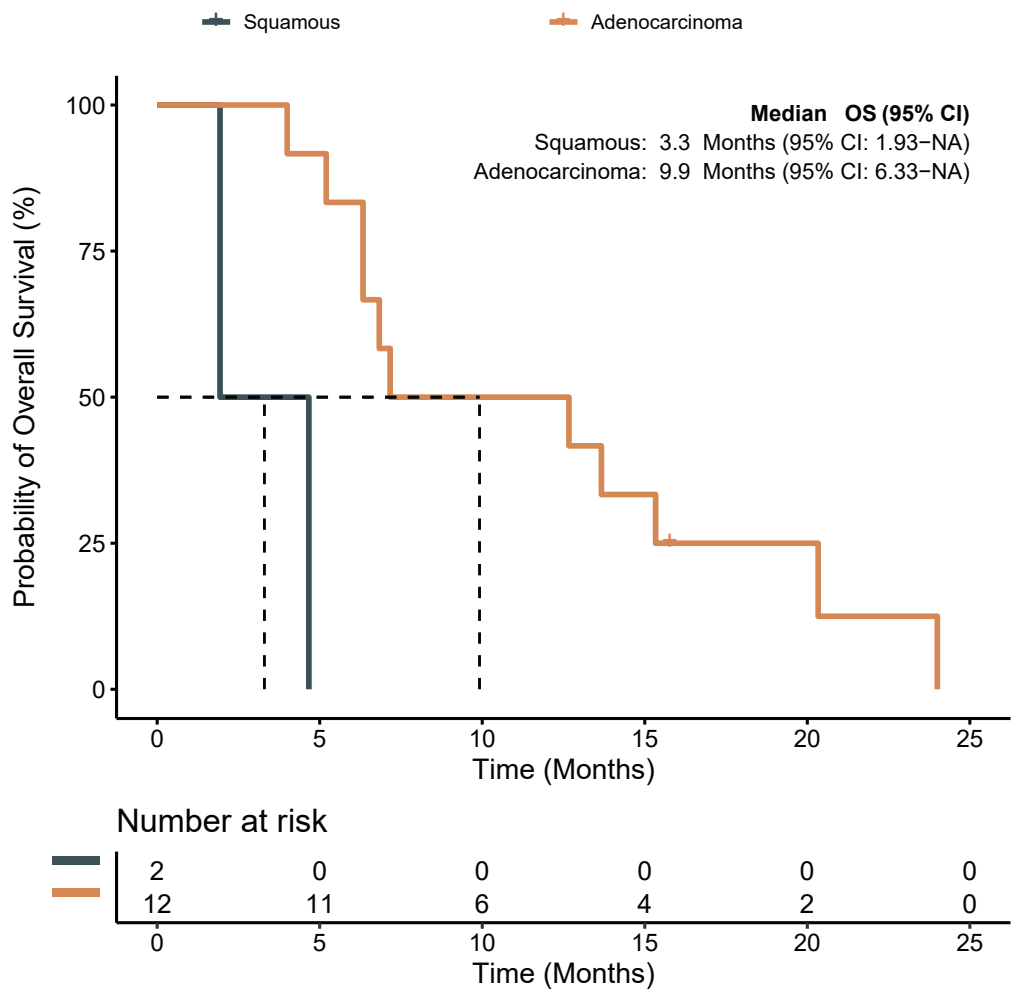

Supplement: Supplementary Figure 2 — Kaplan-Meier survival analysis in patients with adenocarcinoma and squamous treated by anlotinib. (A) Plots of progression-free survival (PFS); (B) Plots of overall survival (OS). [file DataSheet_2.pdf]

(A)

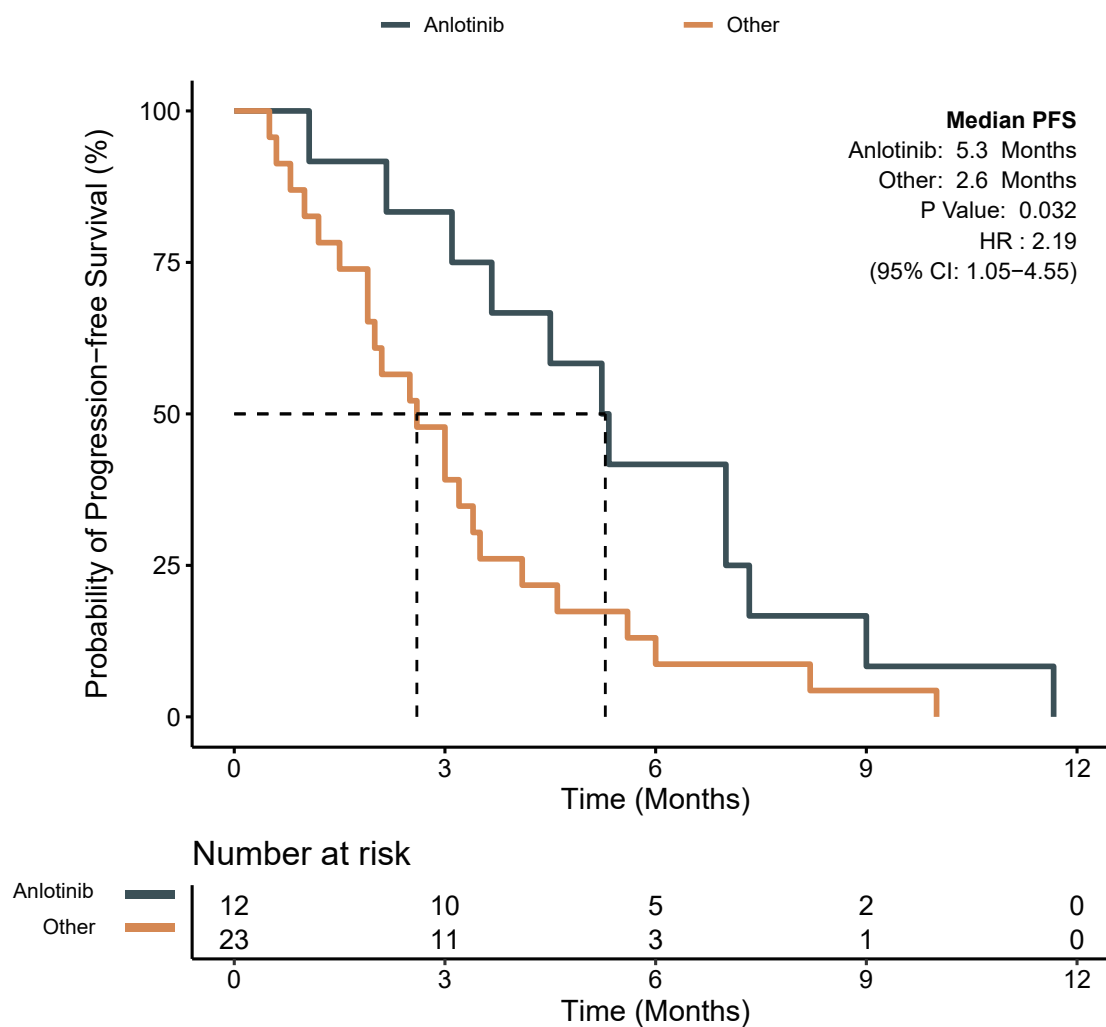

(B)

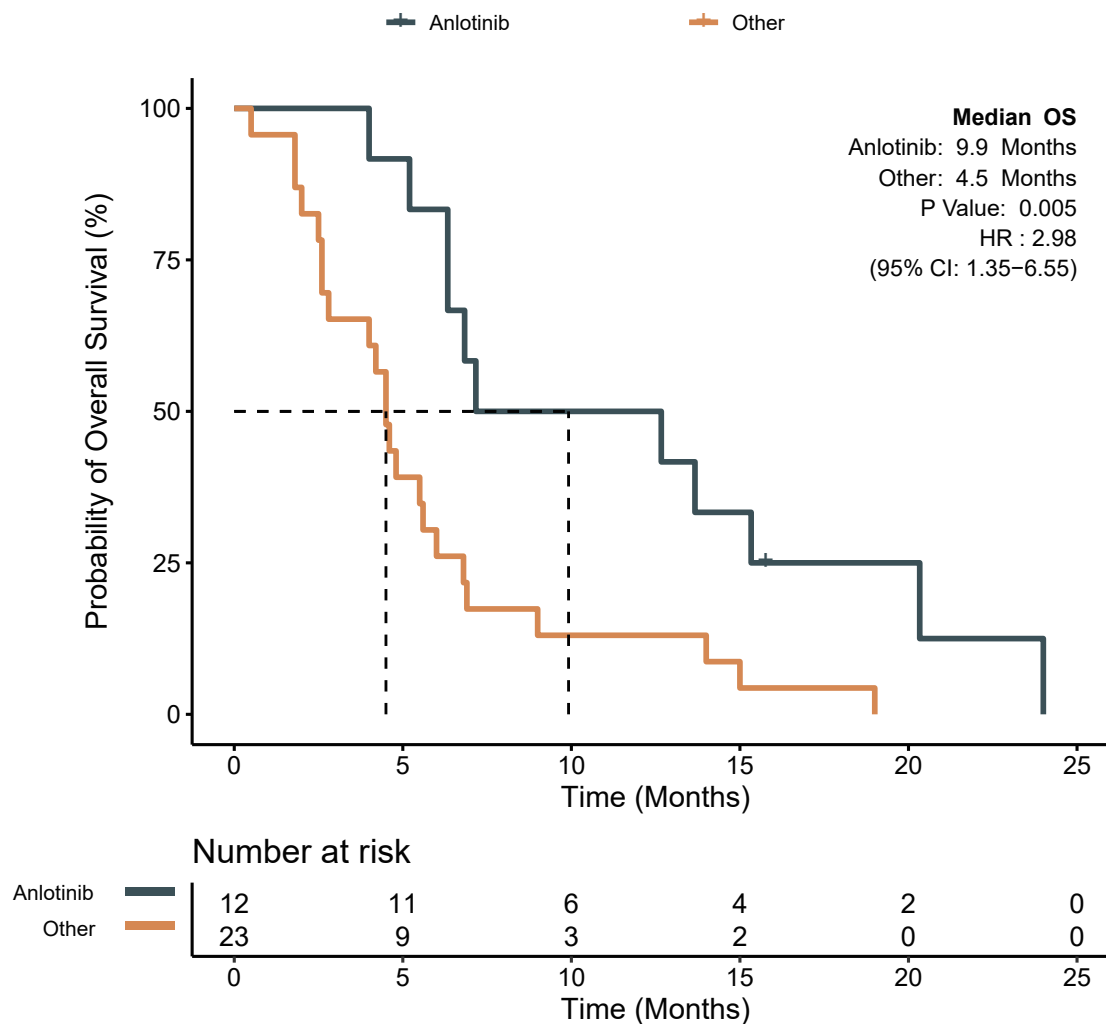

Supplement: Supplementary Figure 3 — Kaplan-Meier survival analysis and comparison of survival between group of anlotinib and other in patients with adenocarcinoma (log-rank test, P < 0.05). (A) Plots of progression-free survival (PFS); (B) Plots of overall survival (OS). [file DataSheet_3.pdf]
