# Supplementary material for: Genomic profiling of non-small cell lung cancer with the rare pulmonary lymphangitic carcinomatosis and clinical outcome of the exploratory anlotinib treatment
Source: Front Oncol. 2022 Oct 17;12:992596. doi: 10.3389/fonc.2022.992596 (PMC9620420; doi:10.3389/fonc.2022.992596)
Supplement: Supplementary file 6 [file Table_1.docx]

Table S1. Panel sets of DNA-based NGS for genomic profiling for 18 patients with PLC.

| **Panel ID** | Panel-1 | Panel-2 | Panel-3 | Panel-4 | Panel-5 | Intersecting of Panel 1-5 |
| --- | --- | --- | --- | --- | --- | --- |
| **Size of Panel (Number of Genes)** | 539 | 425 | 688 | 727 | 645 | 274 |
| **Samples based on Panel** | 3 | 7 | 3 | 1 | 4 | 18 |
| **Gene list** | \| A2M \| \| --- \| \| ABCB1 \| \| ABL1 \| \| ABRAXAS1 \| \| ACTL6A \| \| ACTL6B \| \| ACVR1B \| \| ACVR2A \| \| ADH1B \| \| AKT1 \| \| AKT2 \| \| AKT3 \| \| ALDH2 \| \| ALK \| \| ALOX12B \| \| AMER1 \| \| APC \| \| APLNR \| \| AR \| \| ARAF \| \| ARFRP1 \| \| ARID1A \| \| ARID1B \| \| ARID2 \| \| ARID5B \| \| ASXL1 \| \| ATG13 \| \| ATG2A \| \| ATG7 \| \| ATM \| \| ATR \| \| ATRX \| \| AURKA \| \| AURKB \| \| AXIN1 \| \| AXIN2 \| \| AXL \| \| B2M \| \| BAK1 \| \| BAP1 \| \| BARD1 \| \| BCL10 \| \| BCL2 \| \| BCL2L1 \| \| BCL2L11 \| \| BCL2L2 \| \| BCL6 \| \| BCOR \| \| BCORL1 \| \| BIRC3 \| \| BLM \| \| BMPR1A \| \| BRAF \| \| BRCA1 \| \| BRCA2 \| \| BRD4 \| \| BRD7 \| \| BRIP1 \| \| BTG1 \| \| BTG2 \| \| BTK \| \| BUB1B \| \| C10orf11 \| \| CALR \| \| CARD11 \| \| CASP8 \| \| CBFB \| \| CBL \| \| CBR3 \| \| CCN6 \| \| CCND1 \| \| CCND2 \| \| CCND3 \| \| CCNE1 \| \| CD274 \| \| CD70 \| \| CD74 \| \| CD79A \| \| CD79B \| \| CDA \| \| CDC42 \| \| CDC73 \| \| CDH1 \| \| CDK12 \| \| CDK4 \| \| CDK6 \| \| CDK8 \| \| CDKN1A \| \| CDKN1B \| \| CDKN1C \| \| CDKN2A \| \| CDKN2B \| \| CDKN2C \| \| CEBPA \| \| CFH \| \| CFHR1 \| \| CHD4 \| \| CHEK1 \| \| CHEK2 \| \| CIC \| \| CIITA \| \| CREBBP \| \| CRKL \| \| CRLF2 \| \| CSF1R \| \| CSF3R \| \| CTCF \| \| CTLA4 \| \| CTNNA1 \| \| CTNNB1 \| \| CUL3 \| \| CUL4A \| \| CXCR4 \| \| CYLD \| \| CYP17A1 \| \| CYP19A1 \| \| CYP2C19 \| \| CYP2C8 \| \| CYP2D6 \| \| CYP2E1 \| \| DAPK1 \| \| DAXX \| \| DDR1 \| \| DDR2 \| \| DHFR \| \| DICER1 \| \| DIS3 \| \| DNMT1 \| \| DNMT3A \| \| DOT1L \| \| DPYD \| \| DYNC2H1 \| \| EED \| \| EGFR \| \| EML4 \| \| EMSY \| \| EP300 \| \| EPAS1 \| \| EPCAM \| \| EPHA2 \| \| EPHA3 \| \| EPHA5 \| \| EPHA7 \| \| EPHB1 \| \| EPHB4 \| \| ERBB2 \| \| ERBB3 \| \| ERBB4 \| \| ERCC1 \| \| ERCC2 \| \| ERCC3 \| \| ERCC4 \| \| ERCC5 \| \| ERG \| \| ERRFI1 \| \| ESR1 \| \| ETV1 \| \| ETV4 \| \| EWSR1 \| \| EXT1 \| \| EXT2 \| \| EZH2 \| \| EZR \| \| FANCA \| \| FANCC \| \| FANCD2 \| \| FANCE \| \| FANCF \| \| FANCG \| \| FANCI \| \| FANCL \| \| FAS \| \| FAT1 \| \| FAT2 \| \| FAT3 \| \| FAT4 \| \| FBXW7 \| \| FGA \| \| FGF10 \| \| FGF12 \| \| FGF14 \| \| FGF19 \| \| FGF23 \| \| FGF3 \| \| FGF4 \| \| FGF6 \| \| FGFR1 \| \| FGFR2 \| \| FGFR3 \| \| FGFR4 \| \| FH \| \| FLCN \| \| FLT1 \| \| FLT3 \| \| FLT4 \| \| FOXA1 \| \| FOXL2 \| \| FOXO1 \| \| FOXP1 \| \| FUBP1 \| \| FYN \| \| FZR1 \| \| GABRA6 \| \| GATA1 \| \| GATA2 \| \| GATA3 \| \| GATA4 \| \| GATA6 \| \| GGH \| \| GLI1 \| \| GNA11 \| \| GNA13 \| \| GNAQ \| \| GNAS \| \| GREM1 \| \| GRIN2A \| \| GRM3 \| \| GSK3B \| \| GSTM1 \| \| GSTP1 \| \| GSTT1 \| \| H3F3A \| \| H3F3B \| \| H3F3C \| \| HDAC1 \| \| HDAC2 \| \| HGF \| \| HIST1H1C \| \| HIST1H2BD \| \| HIST1H3A \| \| HIST1H3B \| \| HIST1H3C \| \| HIST1H3D \| \| HIST1H3E \| \| HIST1H3G \| \| HIST1H3H \| \| HIST1H3I \| \| HIST1H3J \| \| HIST2H3D \| \| HIST3H3 \| \| HLA-A \| \| HLA-B \| \| HLA-C \| \| HLA-DQB1 \| \| HNF1A \| \| HNF1B \| \| HRAS \| \| HSD3B1 \| \| HUWE1 \| \| ID3 \| \| IDH1 \| \| IDH2 \| \| IFNGR1 \| \| IFNGR2 \| \| IGF1 \| \| IGF1R \| \| IGF2 \| \| IGFN1 \| \| IKBKE \| \| IKZF1 \| \| IL6R \| \| IL6ST \| \| IL7R \| \| INHBA \| \| INPP4B \| \| INSR \| \| IRF2 \| \| IRF4 \| \| IRS1 \| \| IRS2 \| \| JAK1 \| \| JAK2 \| \| JAK3 \| \| JUN \| \| KDM5A \| \| KDM5C \| \| KDM6A \| \| KDR \| \| KEAP1 \| \| KEL \| \| KIT \| \| KLF4 \| \| KLHL6 \| \| KMT2C \| \| KRAS \| \| LATS1 \| \| LATS2 \| \| LIG3 \| \| LMO1 \| \| LRP1B \| \| LTK \| \| LYN \| \| MAF \| \| MAP2K1 \| \| MAP2K2 \| \| MAP2K4 \| \| MAP3K1 \| \| MAP3K13 \| \| MAPK1 \| \| MAPK3 \| \| MAX \| \| MCL1 \| \| MDM2 \| \| MDM4 \| \| MED12 \| \| MEF2B \| \| MEN1 \| \| MERTK \| \| MET \| \| MITF \| \| MKNK1 \| \| MLH1 \| \| MPL \| \| MRE11 \| \| MSH2 \| \| MSH3 \| \| MSH6 \| \| MST1R \| \| MTAP \| \| MTHFR \| \| MTOR \| \| MUC16 \| \| MUTYH \| \| MYB \| \| MYC \| \| MYCL \| \| MYCN \| \| MYD88 \| \| NBN \| \| NCOR1 \| \| NF1 \| \| NF2 \| \| NFE2L1 \| \| NFE2L2 \| \| NFKBIA \| \| NKX2-1 \| \| NKX3-1 \| \| NOTCH1 \| \| NOTCH2 \| \| NOTCH3 \| \| NOTCH4 \| \| NPM1 \| \| NQO1 \| \| NRAS \| \| NSD1 \| \| NSD2 \| \| NSD3 \| \| NT5C2 \| \| NTHL1 \| \| NTRK1 \| \| NTRK2 \| \| NTRK3 \| \| NUP93 \| \| NUTM1 \| \| P2RY8 \| \| PAK1 \| \| PAK3 \| \| PAK5 \| \| PALB2 \| \| PARP1 \| \| PARP2 \| \| PARP3 \| \| PAX5 \| \| PBRM1 \| \| PDCD1 \| \| PDCD1LG2 \| \| PDE4D \| \| PDGFRA \| \| PDGFRB \| \| PDK1 \| \| PHOX2B \| \| PIGR \| \| PIK3C2B \| \| PIK3C2G \| \| PIK3C3 \| \| PIK3CA \| \| PIK3CB \| \| PIK3CG \| \| PIK3R1 \| \| PIK3R2 \| \| PIM1 \| \| PLCG2 \| \| PLK1 \| \| PMS1 \| \| PMS2 \| \| POLD1 \| \| POLE \| \| PPARG \| \| PPP2R1A \| \| PRDM1 \| \| PREX2 \| \| PRKAR1A \| \| PRKCI \| \| PRKDC \| \| PRKN \| \| PRSS1 \| \| PTCH1 \| \| PTEN \| \| PTK2 \| \| PTPN11 \| \| PTPRB \| \| PTPRD \| \| PTPRO \| \| QKI \| \| RAC1 \| \| RAC2 \| \| RAD17 \| \| RAD21 \| \| RAD50 \| \| RAD51 \| \| RAD51B \| \| RAD51C \| \| RAD51D \| \| RAD52 \| \| RAD54L \| \| RAF1 \| \| RARA \| \| RB1 \| \| RBM10 \| \| RECQL4 \| \| REL \| \| RET \| \| RHEB \| \| RHOA \| \| RICTOR \| \| RNF43 \| \| ROS1 \| \| RPS6KA3 \| \| RPTOR \| \| RRM1 \| \| RSPO2 \| \| RUNX1 \| \| RXRA \| \| SBDS \| \| SDC4 \| \| SDHA \| \| SDHAF2 \| \| SDHB \| \| SDHC \| \| SDHD \| \| SERPINB3 \| \| SERPINB4 \| \| SETD2 \| \| SF3B1 \| \| SGK1 \| \| SH2D1A \| \| SLC34A2 \| \| SLCO1B1 \| \| SLX4 \| \| SMAD2 \| \| SMAD3 \| \| SMAD4 \| \| SMARCA1 \| \| SMARCA2 \| \| SMARCA4 \| \| SMARCB1 \| \| SMARCC1 \| \| SMARCC2 \| \| SMARCD1 \| \| SMARCE1 \| \| SMO \| \| SNCAIP \| \| SOCS1 \| \| SOD2 \| \| SOS1 \| \| SOX10 \| \| SOX17 \| \| SOX2 \| \| SOX9 \| \| SPEN \| \| SPOP \| \| SPTA1 \| \| SRC \| \| SRSF2 \| \| STAG2 \| \| STAT1 \| \| STAT2 \| \| STAT3 \| \| STAT4 \| \| STAT5A \| \| STAT5B \| \| STAT6 \| \| STK11 \| \| SUFU \| \| SUZ12 \| \| SYK \| \| TBX3 \| \| TCF7L2 \| \| TEK \| \| TENT5C \| \| TERT \| \| TET1 \| \| TET2 \| \| TFG \| \| TGFBR1 \| \| TGFBR2 \| \| THADA \| \| TIPARP \| \| TMEM127 \| \| TMEM173 \| \| TMPRSS2 \| \| TNFAIP3 \| \| TNFRSF11A \| \| TNFRSF14 \| \| TNFSF11 \| \| TOP1 \| \| TOP2A \| \| TP53 \| \| TP63 \| \| TPMT \| \| TRAF7 \| \| TSC1 \| \| TSC2 \| \| TSHR \| \| TYMS \| \| TYRO3 \| \| U2AF1 \| \| UGT1A1 \| \| UMPS \| \| VEGFA \| \| VEGFB \| \| VHL \| \| VTCN1 \| \| WAS \| \| WNT10A \| \| WNT10B \| \| WNT7B \| \| WRN \| \| XBP1 \| \| XIAP \| \| XPA \| \| XPC \| \| XPO1 \| \| XRCC1 \| \| XRCC2 \| \| YAP1 \| \| YES1 \| \| ZNF217 \| \| ZNF703 \| | ABCB1(MDR1)  ABCC2(MRP2)  ADH1A  ADH1B  ADH1C  AIP  AKT3  ALDH2  ALK  AMER1  APC  AR  ARAF  ARID1A  ARID1B  ARID2  ARID5B  ASCL4  ASXL1  ATF1  CDC73  PRKACA  PRKACG  PRKAR1A  PRKCI  PRKDC  PRSS3  PTK2  PTPN11  PTPN13  PTPRD  QKI  RAC1  RAC3  RAD50  RAD51  RAD51B  RAD51C  RAD51D  RAD54L  RAF1  RARA  RARG  RASGEF1A  RB1  RECQL4  RELN  RET  RHOA  RICTOR  RNF43  ROS1  SOX14  SOX2  SOX21  SPOP  SPRY4  SRY  STAG2  STAT3  STK11  STMN1  STT3A  SUFU  TAP1  TAP2  TEK  TEKT4  TERC  TERT  TET2  TGFBR2  THADA  TMEM127  TMPRSS2  TNFAIP3  TNFRSF11A  TNFRSF14  TNFRSF19  TNFSF11  TOP1  TOP2A  TP53  TP63  TPMT  ERCC2  IDH2  MTOR  ABCB4  CDH1  ERCC3  IFNG  MUTYH  CDK10  ERCC4  IFNGR1  MYC  CDK12  ERCC5  IGF1R  CDK4  ESR1  IGF2  MYCN  CDK6  ETV1  IKBKE  MYD88  PRSS1  SRC  CDK8  AKT1  CDKN1A  ETV6  IL7R  NAT1  PTCH1  AKT2  CDKN1B  EWSR1  INPP4B  NBN  PTEN  CDKN1C  CDKN2A  CDKN2B  CDKN2C  CEBPA  CEP57  CHD4  CHEK1  CHEK2  CREBBP  CRKL  CSF1R  CTCF  CTLA4  ETV4  EXT1  EXT2  EZH2  FANCA  FANCC  FANCD2  FANCE  FANCF  FANCG  FANCI  FANCL  FANCM  FAT1  FBXW7  FGF19  FGFR1  FGFR2  FGFR3  FGFR4  FH  FLCN  FLT1(VEGFR1)  FLT3  FLT4  FOXA1  IKZF1  IRF2  JAK1  JAK2  JAK3  JARID2  JUN  KDM5A  KDM6A  KDR(VEGFR2)  KEAP1  KIF1B  KIF5B  KIT  KITLG  KLLN  KMT2A(MLL)  KMT2B  KMT2C  KMT2D(MLL2)  KRAS  LHCGR  LMO1  LRP1B  LYN  LZTR1  MYCL  MYH9  NCOR1  NF1  NF2  NFE2L2  NFKBIA  NKX2-1  NKX2-4  NOTCH1  NOTCH2  NOTCH3  NPM1  NQO1  NRAS  NRG1  NSD1  NTRK1  NTRK2  NTRK3  PAK3  PALB2  PALLD  PARK2  PARP1  PARP2  PAX5  ATIC  CTNNB1  ATM  ATR  ATRX  AURKA  AURKB  AXIN2  AXL  B2M  BAD  BAI3  CUL3  CUX1  CXCR4  CYLD  CYP19A1  CYP2A13  CYP2A6  CYP2A7  CYP2B6*6  CYP2C19*2  BAK1  BAP1  BARD1  BAX  BCL2  BIRC3  CYP2D6  CYP3A5  DAXX  FRG1  GATA2  GATA3  MAP3K1  MAP3K4  CYP2C9*3  FOXP1  MAP2K1(MEK1)  PBRM1  RPTOR  TSC1  MAP2K2(MEK2)  PDCD1(PD1)  RRM1  TSC2  CYP3A4*4  GATA1  MAP2K4  PDCD1LG2(PD-L2)  RUNX1  TSHR  PDE11A  RUNX1T1  TTF1  PDGFRA  SBDS  TUBB3  BCL2L11(BIM)  DDR2  GATA4  MAP4K3  PDGFRB  SDC4  TUBB4A  BCR  DENND1A  GATA6  MAX  PDK1  SDHA  TUBB4B  DHFR  GNA11  MCL1  PGR  SDHB  TUBB6  BLM  DICER1  GNAQ  MDM2  PHOX2B  BMPR1A  DLL3  GNAS  MDM4  PIK3C3  BRAF  DNMT3A  GRIN2A  MECOM  PIK3CA  BRCA1  DPYD  GRM3  MED12  PIK3R1  SDHC  TYMS  SDHD  U2AF1  SF3B1  VHL  SGK1  WAS  SLC34A2  WISP3  SLC3A2  WRN  SLC7A8  WT1  SMAD2  XPA  SMAD3  XPC  SMAD4  XRCC1  SMAD7  YAP1  SMARCA4  ZNF2  UGT1A1  VAMP2  SETD2  VEGFA  9-Sep  SETBP1  BRCA2  DUSP2  BRD4  EGFR  BRIP1  EML4  BTG2  EP300  BTK  EPAS1  BUB1B  EPCAM  c11orf30  EPHA2  CASP8  EPHA3  CBL  EPHA5  CBLB  EPHB2  GRM8  MEF2B  PIK3R2  CCND1  CCNE1  CD274(PD-L1)  CD74  CDA  ERBB2(HER2)  ERBB2IP  ERBB3  ERBB4  ERCC1  GSTM1  GSTM4  GSTM5  GSTP1  GSTT1  HDAC2  HDAC9  HGF  HLA-A  HNF1A  HNF1B  HRAS  HSD3B1  IDH1  MEN1  PKHD1  MET  PLAG1  MGMT  PLK1  MITF  PMS1  MLH1  PMS2  MLH3  POLD1  MLLT1  POLD3  MLLT3  POLE  MLLT4  POLH  MPL  POT1  MRE11A  PPARD  MSH2  PPP2R1A  MSH6  PRDM1  MTHFR  PRF1  SMARCB1  SMO  SOS1  SOX1  ZNF217  ZNF703 | ABCB1  ABCG2  ABL1  ABRAXAS1  ACSL3  ACVR1  ACVR2A  ACYP2  ADGRA2  AFF4  AJUBA  AKT1  AKT2  AKT3  ALK  AMER1  APC  APOB  AR  ARAF  ARID1A  ARID1B  ARID2  ASXL1  ATAD2  ATF1  ATM  ATR  ATRX  AURKA  AURKB  AXIN1  AXIN2  AXL  B2M  BABAM2  BACH1  BAP1  BARD1  BCL2  BCL2A1  BCL2L1  BCL6  BCOR  BCR  BIRC2  BIRC3  BLM  BMPR1A  BRAF  BRCA1  BRCA2  BRCC3  BRD4  BRF1  BRIP1  BTK  C8orf34  CARD11  CARM1  CASP8  CASR  CBL  CBLB  CBR3  CBX4  CCDC6  CCNA2  CCND1  CCND2  CCND3  CCNE1  CD274  CD276  CD74  CD79B  CDC27  CDC42  CDC73  CDH1  CDH9  CDK12  CDK4  CDK6  CDK8  CDKN1A  CDKN1B  CDKN1C  CDKN2A  CDKN2B  CDKN2C  CDRT4  CDX2  CEBPA  CETN2  CFTR  CHD1  CHEK1  CHEK2  CIC  CLK2  COL11A1  COL22A1  COP1  CREB1  CREBBP  CRKL  CSDE1  CSF1R  CSMD3  CTCF  CTLA4  CTNNA1  CTNNB1  CTNND2  CUL3  CUL4A  CUL4B  CXCR4  CYLD  CYP11B1  CYP17A1  CYP19A1  CYP2C8  CYP2D6  DAXX  DCUN1D1  DDB2  DDR1  DDR2  DICER1  DIS3  DMC1  DNMT3A  DNTT  DOCK2  DOT1L  DPYD  DSCAM  DUSP4  DUT  DYNC2H1  E2F3  EDC4  EGFR  EIF1AX  EIF4A2  ELAC2  ELF3  ELOC  EME1  EME2  EML4  EMSY  EP300  EPCAM  EPHA2  EPHA3  EPHA4  EPHB1  EPPK1  ERBB2  ERBB3  ERBB4  ERCC1  ERCC2  ERCC3  ERCC4  ERCC5  ERCC6  ERF  ERG  ERRFI1  ESR1  ETV1  ETV4  ETV5  ETV6  EWSR1  EXO1  EXOC2  EXT1  EXT2  EZH1  EZH2  EZR  FAM135B  FAN1  FANCA  FANCB  FANCC  FANCD2  FANCE  FANCF  FANCG  FANCI  FANCL  FANCM  FAT1  FAT2  FAT3  FAT4  FBXW7  FCGR2B  FCGR3A  FGD4  FGF10  FGF12  FGF14  FGF19  FGF2  FGF3  FGF4  FGF6  FGFR1  FGFR2  FGFR3  FGFR4  FH  FLCN  FLI1  FLNA  FLT1  FLT3  FLT4  FOXA1  FOXL2  FOXO1  FOXP1  FRAS1  FUBP1  FYN  G6PC  GAB2  GABRA6  GALNT12  GATA1  GATA2  GATA3  GATA4  GATA6  GEN1  GGH  GID4  GLI1  GNA11  GNAQ  GNAS  GPS2  GRB7  GREM1  GRIN2A  GRM3  GSK3B  GSTP1  H1-2  H2AX  H2BC5  H3-3A  H3-3B  H3-4  H3C1  H3C10  H3C11  H3C13  H3C14  H3C2  H3C3  H3C4  H3C6  H3C7  H3C8  HDAC1  HGF  HLA-A  HLA-B  HNF1A  HOXB13  HRAS  HSD17B4  HSD3B1  HSP90AA1  HSPA4  ICOSLG  ID3  IDH1  IDH2  IFNGR1  IGF1  IGF1R  IGF2  IGF2R  IKBKE  IKZF1  IL10  IL7R  INHA  INHBA  INPP4A  INPP4B  INSR  IRF2  IRF4  IRS2  JAK1  JAK2  JAK3  JMJD1C  JUN  KDM5C  KDM6A  KDR  KEAP1  KIAA1549  KIF1B  KIF5B  KIT  KLF6  KLHL6  KLLN  KMT2A  KMT2B  KMT2C  KMT2D  KMT5A  KNSTRN  KRAS  LAMA2  LATS1  LATS2  LHCGR  LIFR  LIG4  LRP1B  LRRK1  LRRK2  LTK  LYN  LZTR1  MALT1  MAP2K1  MAP2K2  MAP2K4  MAP3K1  MAP3K13  MAP3K14  MAP3K4  MAP4K3  MAPK1  MAPK3  MAPKAP1  MAX  MB21D2  MC1R  MCL1  MDC1  MDH2  MDM2  MDM4  MECOM  MED12  MEF2B  MEN1  MERTK  MET  MGA  MGMT  MITF  MKNK1  MLH1  MLH3  MMS19  MPL  MRE11  MS4A1  MSH2  MSH3  MSH4  MSH5  MSH6  MSI1  MSI2  MST1  MST1R  MTAP  MTDH  MTHFR  MTOR  MTRR  MUC16  MUC6  MUS81  MUTYH  MYB  MYC  MYCL  MYCN  MYD88  MYOD1  MYSM1  NABP2  NBN  NCOA2  NCOA3  NCOA4  NCOR1  NCOR2  NEGR1  NEIL2  NF1  NF2  NFE2L2  NFKB1  NFKBIA  NHEJ1  NKX2-1  NKX3-1  NLRP1  NOTCH1  NOTCH2  NOTCH3  NOTCH4  NPM1  NQO1  NR4A3  NRAS  NSD1  NSD2  NSD3  NT5C2  NTHL1  NTRK1  NTRK2  NTRK3  NUDT18  NUF2  NUTM1  NYAP2  PAK1  PAK5  PALB2  PARP1  PARP2  PARP3  PARP4  PAX5  PAX8  PBRM1  PBX1  PCDH9  PDCD1  PDCD1LG2  PDGFRA  PDGFRB  PDK1  PGR  PHF6  PHOX2B  PIK3CA  PIK3CB  PIK3CG  PIK3R1  PIK3R2  PIK3R3  PIM1  PLAG1  PLCG2  PLK1  PLK2  PLXNA1  PMAIP1  PMS1  PMS2  PNPLA3  PNRC1  POLD1  POLE  POLG  POLH  POLM  POLN  POLQ  POT1  POU5F1  PPARG  PPM1D  PPP2R1A  PPP2R2A  PPP4R2  PPP6C  PRDM1  PRDM14  PREX2  PRKAR1A  PRKCI  PRKD1  PRKDC  PRKN  PRPF40B  PRSS1  PTCH1  PTCH2  PTEN  PTGIS  PTP4A1  PTPN11  PTPRD  PTPRO  PTPRS  PTPRT  QKI  RAB35  RAC1  RAC2  RAD21  RAD50  RAD51  RAD51B  RAD51C  RAD51D  RAD52  RAD54B  RAD54L  RAF1  RARA  RASA1  RB1  RBBP8  RBM10  RECQL  RECQL4  REEP5  REL  RET  RFC4  RHEB  RHOA  RICTOR  RIT1  RNF43  ROS1  RPS6KA3  RPS6KA4  RPS6KB2  RRAGC  RRAS  RRAS2  RSPO2  RTEL1  RUFY4  RUNX1  RXRA  RYBP  RYR2  RYR3  SCG5  SDC4  SDHA  SDHAF2  SDHB  SDHC  SDHD  SEMA3C  SESN1  SESN2  SESN3  SETD2  SF3B1  SGK1  SH2B3  SH2D1A  SHOC2  SHPRH  SHQ1  SIPA1  SLC28A3  SLC34A2  SLC45A3  SLC7A8  SLCO1B1  SLX1A  SLX4  SMAD2  SMAD3  SMAD4  SMARCA1  SMARCA4  SMARCB1  SMARCD1  SMO  SMYD3  SNCAIP  SOCS1  SOD2  SOS1  SOX10  SOX17  SOX2  SOX4  SOX9  SPEN  SPINK1  SPOP  SPOPL  SPRED1  SRC  SRSF2  STAG1  STAG2  STAT3  STAT5A  STAT5B  STK11  STK19  STK40  SUFU  SUZ12  SYK  TAF15  TAF1L  TAP1  TAP2  TBL1XR1  TBX3  TCF3  TCF4  TCF7L2  TEK  TERT  TET1  TET2  TFE3  TGFBR1  TGFBR2  TIPARP  TMEM127  TMPRSS2  TNFAIP3  TNFRSF14  TNFSF11  TOP1  TOP3A  TOPBP1  TP53  TP53BP1  TP63  TPM3  TRAF2  TRAF7  TRRAP  TSC1  TSC2  TSHR  TUBB3  TYMS  U2AF1  UGT1A1  UMPS  UNC5D  UPF1  USP6  VEGFA  VHL  VTCN1  WEE1  WRN  WT1  WWTR1  XIAP  XPA  XPC  XPO1  XRCC1  XRCC2  XRCC3  YAP1  YES1  YWHAZ  ZBTB16  ZFHX3  ZFHX4  ZMYM3  ZNF2  ZNF217  ZNF703  ZNF770  ZNRF3  ZRSR2 | ABCA9  ABCB1  ABCC3  ABCC5  ABL1  ABL2  ACVR1  ACVR1B  ADH1B  AGO2  AKT1  AKT2  AKT3  ALDH2  ALK  ALOX12B  AMER1  ANKRD11  APC  APOE  AR  ARAF  ARFRP1  ARID1A  ARID1B  ARID2  ARID5B  ARV1  ASAP2  ASPSCR1  ASXL1  ASXL2  ATF1  ATIC  ATM  ATR  ATRX  AURKA  AURKB  AXIN1  AXIN2  AXL  B2M  BABAM1  BAP1  BARD1  BBC3  BCL10  BCL2  BCL2L1  BCL2L11  BCL2L2  BCL6  BCOR  BCORL1  BCR  BIRC3  BLM  BMPR1A  BNIP2  BRAF  BRCA1  BRCA2  BRD4  BRIP1  BTG1  BTG2  BTK  BUB1  BUB3  C11orf30  CALR  CARD11  CARM1  CASP7  CASP8  CBFB  CBL  CBR3  CCDC138  CCDC6  CCND1  CCND2  CCND3  CCNE1  CD22  CD274  CD276  CD44  CD70  CD74  CD79A  CD79B  CDA  CDC42  CDC73  CDCP2  CDH1  CDK12  CDK4  CDK6  CDK8  CDKN1A  CDKN1B  CDKN2A  CDKN2B  CDKN2C  CEBPA  CENPA  CFTR  CHD2  CHD4  CHEK1  CHEK2  CIC  CNOT1  COL4A3BP  CPSF7  CREB1  CREB5  CREBBP  CRKL  CRLF2  CRTC1  CSDE1  CSF1R  CSF3R  CTCF  CTLA4  CTNNA1  CTNNB1  CUL3  CUL4A  CXCR4  CYBA  CYLD  CYP17A1  CYP19A1  CYP1B1  CYP2C19  CYP2C8  CYP2D6  CYP4F12  CYSLTR2  DAXX  DCUN1D1  DDR1  DDR2  DENND1C  DGUOK  DHFR  DHX30  DICER1  DIS3  DNAH7  DNAJB1  DNMT1  DNMT3A  DNMT3B  DOPEY2  DOT1L  DPYD  DROSHA  DSCAM  DUSP4  DYNC2H1  E2F3  EED  EGFL7  EGFR  EIF1AX  EIF4A2  EIF4E  ELF3  EML4  EP300  EPAS1  EPCAM  EPHA3  EPHA5  EPHA7  EPHB1  EPHB4  EPS8  ERBB2  ERBB3  ERBB4  ERCC1  ERCC2  ERCC3  ERCC4  ERCC5  ERF  ERG  ERRFI1  ESR1  ETV1  ETV4  ETV5  ETV6  EWSR1  EXOSC1  EZH1  EZH2  EZR  FAM175A  FAM46C  FAM58A  FANCA  FANCC  FANCD2  FANCE  FANCF  FANCG  FANCL  FAS  FAT1  FBXW7  FDPS  FGF10  FGF12  FGF14  FGF19  FGF23  FGF3  FGF4  FGF6  FGFR1  FGFR1OP  FGFR2  FGFR3  FGFR4  FH  FLCN  FLI1  FLT1  FLT3  FLT4  FOCAD  FOXA1  FOXL2  FOXO1  FOXP1  FRS2  FUBP1  FUS  FYN  FYTTD1  GABRA6  GALNT12  GALNT14  GATA1  GATA2  GATA3  GATA4  GATA6  GID4  GLI1  GMCL1  GNA11  GNA13  GNAQ  GNAS  GNG12  GPR124  GPS2  GREM1  GRIN2A  GRM3  GSK3B  GSTA1  GSTP1  H3F3A  H3F3B  H3F3C  HAPLN1  HDAC1  HECTD2  HGF  HIST1H1C  HIST1H2BD  HIST1H3A  HIST1H3B  HIST1H3C  HIST1H3D  HIST1H3E  HIST1H3F  HIST1H3G  HIST1H3H  HIST1H3I  HIST1H3J  HIST2H3C  HIST2H3D  HIST3H3  HLA-A  HLA-B  HLA-C  HNF1A  HNF4G  HNRNPH1  HOXB13  HRAS  HSD3B1  HSF2BP  HSP90AA1  ICOSLG  ID3  IDH1  IDH2  IFNGR1  IGF1  IGF1R  IGF2  IKBKE  IKZF1  IL10  IL6  IL7R  INHA  INHBA  INPP4A  INPP4B  INPPL1  INSR  IRF2  IRF4  IRS1  IRS2  JAK1  JAK2  JAK3  JAZF1  JUN  KAT6A  KDM5A  KDM5C  KDM6A  KDR  KEAP1  KEL  KIAA1429  KIAA1549  KIAA1919  KIF5B  KIT  KLF4  KLHL6  KMT2A  KMT2B  KMT2C  KMT2D  KNSTRN  KPNB1  KRAS  KRT26  LATS1  LATS2  LMO1  LOC494141  LRP1B  LSM3  LTK  LYN  LZTR1  MAF  MAGI2  MALT1  MAML2  MAP2K1  MAP2K2  MAP2K4  MAP3K1  MAP3K13  MAP3K14  MAP4K3  MAPK1  MAPK3  MAPKAP1  MARS  MAX  MCL1  MDC1  MDM2  MDM4  MED12  MEF2B  MEN1  MERTK  MET  MGA  MGST2  MICU3  MITF  MKLN1  MKNK1  MLH1  MNDA  MPL  MRE11  MRE11A  MRPL1  MSH2  MSH3  MSH6  MSI1  MSI2  MST1  MST1R  MTAP  MTHFR  MTOR  MTR  MTRR  MTTP  MTUS1  MUTYH  MYB  MYC  MYCL  MYCN  MYD88  MYOD1  NAA25  NARG2  NBN  NCOA3  NCOA4  NCOR1  NDRG1  NEGR1  NF1  NF2  NFE2L2  NFIB  NFKBIA  NKX2  NKX3  NMD3  NOTCH1  NOTCH2  NOTCH3  NOTCH4  NPM1  NQO1  NR4A3  NRAS  NSD1  NT5C2  NTHL1  NTRK1  NTRK2  NTRK3  NUF2  NUP93  NUTM1  OR7C1  P2RY8  PAK1  PAK3  PAK7  PALB2  PARK2  PARP1  PARP2  PARP3  PAX5  PAX8  PBRM1  PBX1  PCNXL4  PDCD1  PDCD1LG2  PDGFRA  PDGFRB  PDK1  PDPK1  PGM2  PGR  PHOX2B  PHTF1  PIK3C2B  PIK3C2G  PIK3C3  PIK3CA  PIK3CB  PIK3CD  PIK3CG  PIK3R1  PIK3R2  PIK3R3  PIM1  PIP5K1A  PLAG1  PLCG2  PLK2  PMAIP1  PMS1  PMS2  PNRC1  POLD1  POLE  POU5F1  PPARG  PPL  PPM1D  PPP2R1A  PPP2R2A  PPP4R2  PPP6C  PPRC1  PRCC  PRDM1  PRDM14  PREX2  PRKAR1A  PRKCI  PRKD1  PRKDC  PRPF8  PRSS1  PRSS8  PTCH1  PTEN  PTP4A1  PTPN11  PTPRD  PTPRO  PTPRS  PTPRT  QKI  RAB35  RAC1  RAC2  RAD21  RAD50  RAD51  RAD51B  RAD51C  RAD51D  RAD52  RAD54L  RAF1  RANBP2  RARA  RASA1  RASEF  RB1  RBM10  RECQL  RECQL4  REL  RET  RFPL3S  RFWD2  RHEB  RHOA  RICTOR  RIT1  RNF43  ROS1  RPS6KA4  RPS6KB2  RPTOR  RRAGC  RRAS  RRAS2  RRM1  RSPO2  RTEL1  RUNX1  RUNX1T1  RXFP2  RXRA  RYBP  SDC4  SDHA  SDHAF2  SDHB  SDHC  SDHD  SESN1  SESN2  SESN3  SETD2  SETD8  SF3B1  SGIP1  SGK1  SH2B3  SH2D1A  SH2D1B  SHB  SHOC2  SHQ1  SLC29A1  SLC29A4  SLC30A5  SLC34A2  SLC45A3  SLC5A8  SLC7A8  SLCO1B3  SLIT1  SLIT2  SLX4  SMAD2  SMAD3  SMAD4  SMARCA4  SMARCB1  SMARCD1  SMC6  SMO  SMYD3  SNCAIP  SND1  SNTG1  SOCS1  SOD2  SOS1  SOX10  SOX17  SOX2  SOX9  SPAG9  SPEN  SPINK1  SPOP  SPRED1  SPTA1  SRC  SRSF2  STAG2  STAMBP  STAT3  STAT4  STAT5A  STAT5B  STK11  STK19  STK38L  STK40  SUCLG2  SUFU  SUZ12  SYK  TACC3  TAF1  TAF15  TAP1  TAP2  TARBP1  TATDN2  TBX3  TCEB1  TCF3  TCF4  TCF7L2  TEC  TEK  TERC  TERT  TET1  TET2  TFE3  TGFBR1  TGFBR2  THOC1  TIAL1  TIMM17A  TIPARP  TM9SF3  TMEM127  TMPRSS2  TNC  TNFAIP3  TNFRSF14  TOP1  TOP2A  TP53  TP53BP1  TP63  TPM3  TPMT  TRAF2  TRAF7  TRIM33  TSC1  TSC2  TSHR  TTC3  TTC30A  TYMS  TYRO3  U2AF1  UBR5  UGT1A1  UGT2B4  UMPS  UPF1  URI1  USP40  VEGFA  VHL  VPS36  VTCN1  WBP11  WDR70  WHSC1  WHSC1L1  WISP3  WT1  WWP1  WWTR1  XIAP  XPC  XPO1  XRCC1  XRCC2  YAP1  YES1  ZBTB2  ZFHX3  ZNF2  ZNF217  ZNF318  ZNF703 | ABCB1  ABCC3  ABL1  ABL2  ACVR1  ACVR1B  AGO2  AKT1  AKT2  AKT3  ALK  ALOX12B  AMER1  ANKRD11  APC  APEX1  AR  ARAF  ARFRP1  ARID1A  ARID1B  ARID2  ARID5B  ASNS  ASXL1  ASXL2  ATIC  ATM  ATR  ATRX  AURKA  AURKB  AXIN1  AXIN2  AXL  B2M  BABAM1  BAP1  BARD1  BBC3  BCL10  BCL2  BCL2L1  BCL2L11  BCL2L2  BCL6  BCOR  BCORL1  BCR  BIRC3  BIRC7  BLM  BMPR1A  BRAF  BRCA1  BRCA2  BRD4  BRIP1  BTG1  BTG2  BTK  C11orf30  C8orf34  CALR  CARD11  CARM1  CASP7  CASP8  CBFB  CBL  CBR3  CCND1  CCND2  CCND3  CCNE1  CD22  CD274  CD276  CD3EAP  CD44  CD70  CD79A  CD79B  CDA  CDC42  CDC73  CDH1  CDK12  CDK4  CDK6  CDK8  CDKN1A  CDKN1B  CDKN2A  CDKN2B  CDKN2C  CEBPA  CENPA  CHD2  CHD4  CHEK1  CHEK2  CIC  CREBBP  CRKL  CRLF2  CSDE1  CSF1R  CSF3R  CTCF  CTLA4  CTNNA1  CTNNB1  CTTN  CUL3  CUL4A  CXCR4  CYLD  CYP17A1  CYP19A1  CYP1B1  CYP2C8  CYP2D6  CYP4B1  CYSLTR2  DAXX  DCUN1D1  DDR1  DDR2  DDX43  DICER1  DIS3  DNAJB1  DNMT1  DNMT3A  DNMT3B  DOT1L  DPYD  DROSHA  DUSP4  DYNC2H1  E2F3  EED  EGFL7  EGFR  EIF1AX  EIF4A2  EIF4E  ELF3  EP300  EPAS1  EPCAM  EPHA2  EPHA3  EPHA5  EPHA7  EPHB1  EPHB4  ERBB2  ERBB3  ERBB4  ERCC1  ERCC2  ERCC3  ERCC4  ERCC5  ERF  ERG  ERRFI1  ESR1  ESR2  ETV1  ETV6  EWSR1  EXT1  EZH1  EZH2  FADD  FAM175A  FAM46C  FAM58A  FANCA  FANCC  FANCD2  FANCE  FANCF  FANCG  FANCI  FANCL  FANCM  FAS  FAT1  FAT3  FBXW7  FCGR2A  FCGR3A  FGF10  FGF12  FGF14  FGF19  FGF23  FGF3  FGF4  FGF6  FGFR1  FGFR2  FGFR3  FGFR4  FH  FLCN  FLT1  FLT3  FLT4  FOXA1  FOXL2  FOXO1  FOXP1  FRS2  FSHR  FUBP1  FYN  GAB2  GABRA6  GALNT12  GATA1  GATA2  GATA3  GATA4  GATA6  GEN1  GGH  GID4  GLI1  GNA11  GNA13  GNAQ  GNAS  GPR124  GPS2  GREM1  GRIN2A  GRM3  GSK3B  GSTA1  GSTM1  GSTP1  H3F3A  H3F3B  H3F3C  HAS3  HDAC1  HDAC6  HGF  HIST1H1C  HIST1H2BD  HIST1H3A  HIST1H3B  HIST1H3C  HIST1H3D  HIST1H3E  HIST1H3F  HIST1H3G  HIST1H3H  HIST1H3I  HIST1H3J  HIST2H3C  HIST2H3D  HIST3H3  HLA-A  HLA-B  HMMR  HNF1A  HOXB13  HRAS  HSD3B1  HSP90AA1  HSPB1  ICOSLG  ID3  IDH1  IDH2  IFNGR1  IGF1  IGF1R  IGF2  IKBKE  IKZF1  IL10  IL1A  IL4  IL7R  IL8  INHA  INHBA  INPP4A  INPP4B  INPPL1  INSR  IRF2  IRF4  IRS1  IRS2  JAK1  JAK2  JAK3  JUN  KAT6A  KDM3B  KDM5A  KDM5C  KDM6A  KDR  KEAP1  KEL  KIT  KLF4  KLHL6  KMT2A  KMT2B  KMT2C  KMT2D  KNSTRN  KRAS  LATS1  LATS2  LIG4  LIMK1  LIN28B  LMO1  LRP1B  LTK  LYN  LZTR1  MAF  MAGI2  MALT1  MAP2K1  MAP2K2  MAP2K4  MAP3K1  MAP3K13  MAP3K14  MAPK1  MAPK3  MAPKAP1  MAX  MCL1  MDC1  MDM2  MDM4  MECOM  MED12  MEF2B  MEN1  MERTK  MET  MGA  MGMT  MITF  MKNK1  MLH1  MLH3  MPL  MRE11A  MSH2  MSH3  MSH6  MSI1  MSI2  MST1  MST1R  MTAP  MTHFR  MTOR  MTRR  MUTYH  MXI1  MYC  MYCL  MYCN  MYD88  MYO3B  MYOD1  NBN  NCOA3  NCOR1  NDRG1  NEGR1  NEIL1  NF1  NF2  NFE2L2  NFKBIA  NKX2-1  NKX3-1  NOS2  NOTCH1  NOTCH2  NOTCH3  NOTCH4  NPM1  NQO1  NQO2  NRAS  NSD1  NT5C2  NTHL1  NTRK1  NTRK2  NTRK3  NUF2  NUP93  OPRM1  P2RY8  PAK1  PAK3  PAK7  PALB2  PARK2  PARP1  PARP2  PARP3  PAX5  PBRM1  PCAP  PDCD1  PDCD1LG2  PDGFRA  PDGFRB  PDK1  PDPK1  PGR  PHB  PHOX2B  PIK3C2B  PIK3C2G  PIK3C3  PIK3CA  PIK3CB  PIK3CD  PIK3CG  PIK3R1  PIK3R2  PIK3R3  PIM1  PLAT  PLCG2  PLK2  PMAIP1  PMS1  PMS2  PNRC1  POLD1  POLE  PON1  PPARG  PPM1D  PPP2R1A  PPP2R2A  PPP4R2  PPP6C  PRDM1  PRDM14  PREX2  PRKAA1  PRKAR1A  PRKCI  PRKD1  PRKDC  PRSS8  PTCH1  PTEN  PTP4A1  PTPN11  PTPRD  PTPRO  PTPRS  PTPRT  QKI  RAB35  RAC1  RAC2  RAD21  RAD50  RAD51  RAD51B  RAD51C  RAD51D  RAD52  RAD54B  RAD54L  RAF1  RANBP2  RARA  RASA1  RB1  RBM10  RECQL  RECQL4  REL  RET  RFWD2  RHBDF2  RHEB  RHOA  RICTOR  RIT1  RNF43  ROS1  RPS6KA4  RPS6KB2  RPTOR  RRAGC  RRAS  RRAS2  RRM1  RSF1  RTEL1  RUNX1  RUNX1T1  RXRA  RYBP  SDHA  SDHAF2  SDHB  SDHC  SDHD  SEMA3C  SESN1  SESN2  SESN3  SETD2  SETD8  SF3B1  SGK1  SH2B3  SH2D1A  SHOC2  SHQ1  SLCO1B1  SLCO1B3  SLIT1  SLIT2  SLX4  SMAD2  SMAD3  SMAD4  SMARCA4  SMARCB1  SMARCD1  SMO  SMYD3  SNCAIP  SOCS1  SOD2  SOS1  SOX10  SOX17  SOX2  SOX4  SOX9  SPEN  SPOP  SPRED1  SPTA1  SRC  SRSF2  STAG2  STAT3  STAT4  STAT5A  STAT5B  STK11  STK19  STK40  SUFU  SUZ12  SYK  TAF1  TAP1  TAP2  TBX3  TCEB1  TCF3  TCF7L2  TDG  TEK  TERC  TERT  TET1  TET2  TGFB1  TGFBR1  TGFBR2  TIPARP  TLR2  TMEM127  TMPRSS2  TNF  TNFAIP3  TNFRSF14  TNFSF11  TOP1  TOP2A  TP53  TP53BP1  TP63  TRAF2  TRAF7  TRRAP  TSC1  TSC2  TSHR  TSHZ2  TSHZ3  TTF1  TXN  TXNRD2  TYMS  TYRO3  U2AF1  UGT1A1  UGT1A4  UMPS  UPF1  VEGFA  VHL  VTCN1  WHSC1  WHSC1L1  WISP3  WT1  WWTR1  XIAP  XPC  XPO1  XRCC1  XRCC2  XRCC3  YAP1  YES1  ZBTB2  ZFHX3  ZNF217  ZNF703 | ABCB1  HNF1A  HRAS  HSD3B1  EGFR  FGF19  FGFR1  MAX  NOTCH1  NOTCH2  NOTCH3  PPP2R1A  PRDM1  SDHA  TEK  SDHB  TERT  CCNE1  CD274  AKT1  AKT2  AKT3  ALK  AMER1  APC  AR  ARAF  ARID1A  ARID1B  ARID2  ASXL1  ATM  ATR  ATRX  AURKA  AURKB  AXIN2  AXL  B2M  BAP1  BARD1  BCL2  CDC73  CDH1  CDK4  CDK6  CDK8  CDK12  CDKN1A  CDKN1B  CDKN2A  CDKN2B  CDKN2C  CEBPA  CHEK1  CHEK2  CREBBP  CRKL  CSF1R  CTCF  CTLA4  EP300  EPCAM  EPHA3  ERBB2  ERBB3  ERBB4  ERCC1  ERCC2  ERCC3  ERCC4  ERCC5  ESR1  ETV1  EWSR1  FGFR2  FGFR3  IDH1  FGFR4  IDH2  FH  IFNGR1  FLCN  IGF1R  IGF2  FLT1  FLT3  IKBKE  FLT4  IKZF1  FOXA1  IL7R  FOXP1  INPP4B  IRF2  JAK1  GATA1  JAK2  GATA2  JAK3  GATA3  GATA4  JUN  GATA6  KDM6A  KDR  KEAP1  GNA11  GNAQ  GNAS  KIT  MCL1  MDM2  MDM4  MED12  MEF2B  MEN1  MET  MITF  MLH1  MPL  MSH2  MSH6  MTHFR  MTOR  MUTYH  MYC  MYCL  MYCN  MYD88  NPM1  NQO1  NRAS  NSD1  NTRK1  NTRK2  NTRK3  PALB2  PRKAR1A  PRKCI  PRKDC  PTCH1  PTEN  PTPN11  PTPRD  SDHC  SDHD  SETD2  SF3B1  SGK1  SMAD2  SMAD3  SMAD4  SMARCA4  SMARCB1  SMO  SOS1  SOX2  SPOP  SRC  TET2  TGFBR2  TMEM127  TMPRSS2  TNFAIP3  TNFRSF14  TOP1  TP53  TP63  TSC1  TSC2  TSHR  TYMS  U2AF1  UGT1A1  VEGFA  VHL  XPC  XRCC1  YAP1  PARP1  QKI  PARP2  RAC1  PAX5  RAD50  CTNNB1  GRIN2A  GRM3  BIRC3  CUL3  EZH2  KMT2C  BLM  GSTP1  BMPR1A  PBRM1  PDCD1  PDCD1LG2  PDGFRA  PDGFRB  PDK1  PHOX2B  PIK3CA  PIK3R1  PIK3R2  RAD51  RAD51B  RAD51C  RAD51D  RAD54L  RAF1  RARA  RB1  RECQL4  RET  RHOA  RICTOR  BRAF  BRCA1  BRCA2  BRD4  CXCR4  CYLD  CYP2D6  FANCA  FANCC  FANCD2  FANCE  KRAS  BRIP1  BTK  CASP8  HGF  HLA-A  CCND1  DPYD  CYP19A1  FANCF  DAXX  FANCG  FANCL  LRP1B  LYN  MAP2K1  MAP2K2  MAP2K4  MAP3K1  NBN  NCOR1  NF1  NF2  NFE2L2  NFKBIA  PMS1  PMS2  RNF43  ROS1  STAG2  STAT3  ZNF217  ZNF703  DDR2  FAT1  DICER1  CBL  DNMT3A  FBXW7  STK11  POLD1  POLE  SUFU  RUNX1 |
