# Supplementary material for: Genomic profiling of non-small cell lung cancer with the rare pulmonary lymphangitic carcinomatosis and clinical outcome of the exploratory anlotinib treatment
Source: Front Oncol. 2022 Oct 17;12:992596. doi: 10.3389/fonc.2022.992596 (PMC9620420; doi:10.3389/fonc.2022.992596)
Supplement: Supplementary file 7 [file Table_2.docx]

Table S2. Comparison of baseline characteristic between patients treated with anlotinib and other strategies.

| Variables | Anlotinib [n(%)] | Other [n(%)] | P value |
| --- | --- | --- | --- |
| No. of Patient | 14 (100%) | 25 (100%) |  |
| Age (years, mean/SD) | 60 (12) | 62 (11) | 0.70 |
| Sex (male) | 9 (64.3%) | 17 (68%) | > 0.9 |
| KPS (>=60) | 10 (71%) | 20 (80%) | 0.70 |
| Smoking history (former) | 5 (35.7%) | 16 (64%) | 0.09 |
| Histological subtype |  |  | 0.60 |
| Adenocarcinoma | 12 (85.7%) | 23 (92%) |  |
| Squamous | 2 (14.3%) | 2 (8%) |  |
| EGFR (mutant) | 4 (28.6%) | 10 (40%) | 1 |
| Genomic profile analyzed | 5 (35.7%) | 13 (52%) | 0.50 |
